# ETS1 suppresses hepatic stellate cell activation and liver fibrosis

**DOI:** 10.1172/jci.insight.195242

**Published:** 2025-11-04

**Authors:** Wonseok Lee, Xiao Liu, Sara Brin Rosenthal, Charlene Miciano, Sadatsugu Sakane, Kanani Hokutan, Debanjan Dhar, Hyun Young Kim, David A. Brenner, Tatiana Kisseleva

**Affiliations:** 1 Department of Medicine,; 2 Department of Surgery, University of California San Diego School of Medicine, La Jolla, California, USA.; 3 College of Pharmacy, Gachon University, Incheon, South Korea.; 4 Center for Computational Biology and Bioinformatics,; 5 Department of Cellular and Molecular Medicine, and; 6 Center for Epigenomics, University of California San Diego School of Medicine, La Jolla, California, USA.; 7 Sanford Burnham Prebys Medical Discovery Institute, La Jolla, California, USA.; 8 College of Pharmacy and; 9 Center for Human Risk Assessment, Dankook University, Cheonan, Chungnam, South Korea.

**Keywords:** Cell biology, Gastroenterology, Hepatology, Extracellular matrix, Fibrosis, Molecular pathology

## Abstract

Chronic liver injury results in activation of quiescent hepatic stellate cells (HSCs) into collagen type I–producing activated HSCs that make the liver fibrotic. We identified ETS1 and ETS2 (ETS1/2) as lineage-specific transcription factors regulating HSC phenotypes. Here, we investigated the role of ETS1/2 in HSCs in liver fibrosis using toxic liver injury models and 3D human liver spheroids. Liver fibrosis was induced in WT and HSC-specific Ets1-KO (*Ets1*^ΔHSC^) and Ets2-KO (*Ets2*^ΔHSC^) mice by administration of CCl_4_ for 6 weeks, followed by cessation of liver injury for 2 weeks. Liver fibrosis was more severe in *Ets1*^ΔHSC^ and to a lesser extent *Ets2*^ΔHSC^ mice compared with WT mice. Regression of liver fibrosis was suppressed only in *Ets1*^ΔHSC^ mice, indicating Ets1 is the predominant isoform maintaining a quiescent-like phenotype in HSCs. Similar results were obtained in a metabolic dysfunction–associated steatohepatitis (MASH) model using 3D human liver spheroids. Knockdown of ETS1 in human HSCs caused upregulation of fibrogenic genes in MASH human liver spheroids and prevented fibrosis regression. ETS1 regulated the quiescent HSC phenotype via the CREB-regulated transcription coactivator 2 (CRTC2)/PGC1α/PPARγ pathway. Knockdown of CRTC2 abrogated PPARγ responses and facilitated HSC activation. These findings suggest that ETS1 may represent a therapeutic target for antifibrotic therapy.

## Abstract 2

ETS1 maintains quiescent phenotype of Hepatic Stellate Cells, suppresses liver fibrosis and facilitates fibrosis regression.

## Graphical abstract

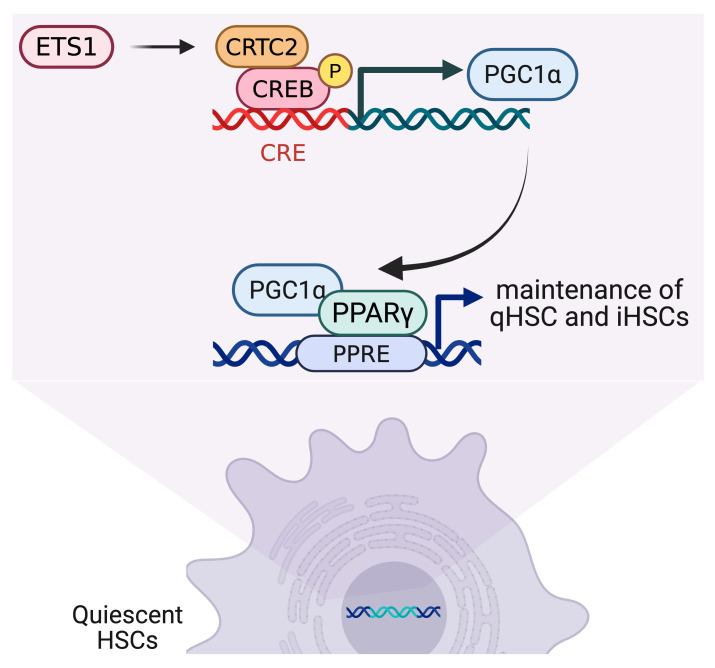

## Introduction

Hepatic fibrosis is caused by chronic toxic liver injury, such as metabolic dysfunction–associated steatohepatitis (MASH) and viral infection (HBV/HCV), which induce inflammation, activation of fibrogenic myofibroblasts, and formation of fibrous scars (1). Hepatic stellate cells (HSCs) are the primary source of myofibroblasts in the fibrotic liver. Under physiological conditions, HSCs exhibit a quiescent phenotype, reside in the space of Disse, and store vitamin A (2). In response to injury, quiescent HSCs activate into collagen type 1–expressing HSCs (aHSCs)/myofibroblasts, generating liver fibrosis (3). Clinical and experimental studies have demonstrated that hepatic fibrosis can regress. Upon cessation of the etiological injury, aHSCs undergo apoptosis or inactivate (iHSCs). iHSCs downregulate the expression of fibrogenic genes and reexpress some but not all quiescence-associated genes such as *Pparg*, *Ngfr*, and *Bambi* (4). Therefore, aHSCs are the primary targets for antifibrotic therapy.

The regulatory mechanism of HSC phenotypes is not well understood. Phenotypic changes in HSCs are regulated on an epigenetic level, which affects DNA accessibility of specific transcription factors (TFs), causing transcriptional activation or repression of their target genes (5). Several TFs are implicated in the maintenance of HSC phenotypes. JUNB/AP-1, ETS1 and ETS2 (ETS1/2), GATA4/6, and IRF1/2 are considered lineage-specific TFs, defined by the ability to regulate lineage determination, responses to stress, and cellular fate (6, 7). Activation of JUNB/AP-1 and TEAD is linked to HSC activation. ETS1/2 and GATA4/6 regulate the quiescent HSC phenotype. Only a few TFs (GATA6, PPARγ, GABRA3) are linked to HSC inactivation. Thus, genetic deletion of GATA6 in HSCs inhibits aHSC inactivation and regression of liver fibrosis.

The E26 transformation specific (ETS) protein family is one of the largest and most evolutionarily conserved families of TFs implicated in organismal development, proliferation, and differentiation (8, 9). Global KO of ETS1/2 results in embryonic lethality (10, 11). ETS1 and ETS2 share significant structural similarity (55%) and are nearly identical in their DNA-binding domains, suggesting potential redundancy in their functions (12). A prior study in cultured rat HSCs demonstrated that ETS1 expression is high in quiescent HSCs and in the early stage of activation but decreases during full activation, suggesting a potential role for ETS1 in maintaining HSC quiescence (13). However, the contribution of ETS1/2 to liver fibrosis in vivo and role in regulating HSC quiescence and fibrosis regression have not been previously investigated.

This study analyzed the role of ETS1/2 in the development and regression of liver fibrosis using HSC-specific Ets1-KO and Ets2-KO mice. We demonstrated that HSC-specific deletion of Ets1 (*Ets1*^ΔHSC^) exacerbated the development of CCl_4_-induced liver fibrosis and prevented fibrosis regression. Deletion of *Ets2* also promoted fibrosis, but to a lesser extent due to compensatory upregulation of *Ets1* in Ets2^ΔHSC^ mice. The role of ETS1 in human HSCs was further assessed using human liver spheroids. Dicer-substrate siRNA–based (dsiRNA-based) knockdown of ETS1 increased activation of human HSCs and accelerated fibrosis in this model of MASH. ETS1 exerts its antifibrotic properties via CRTC2/PGC1α-dependent activation of PPARγ, a known regulator of quiescent and inactivated HSC phenotypes (6). CREB-regulated transcription coactivator 2 (CRTC2) was identified as a direct target of ETS1 that regulates PPARγ activity. Knockdown of ETS1 or CRTC2 suppressed PPARγ expression and increased HSC activation. Collectively, this study demonstrated that activation of ETS1 in HSCs prevented the development of hepatic fibrosis and promoted fibrosis resolution via the CRTC2/PPARγ signaling axis. ETS1 may represent a therapeutic target for antifibrotic therapy.

## Results

### MASH activates human HSCs and downregulates expression of Ets1.

**Figure 1:**
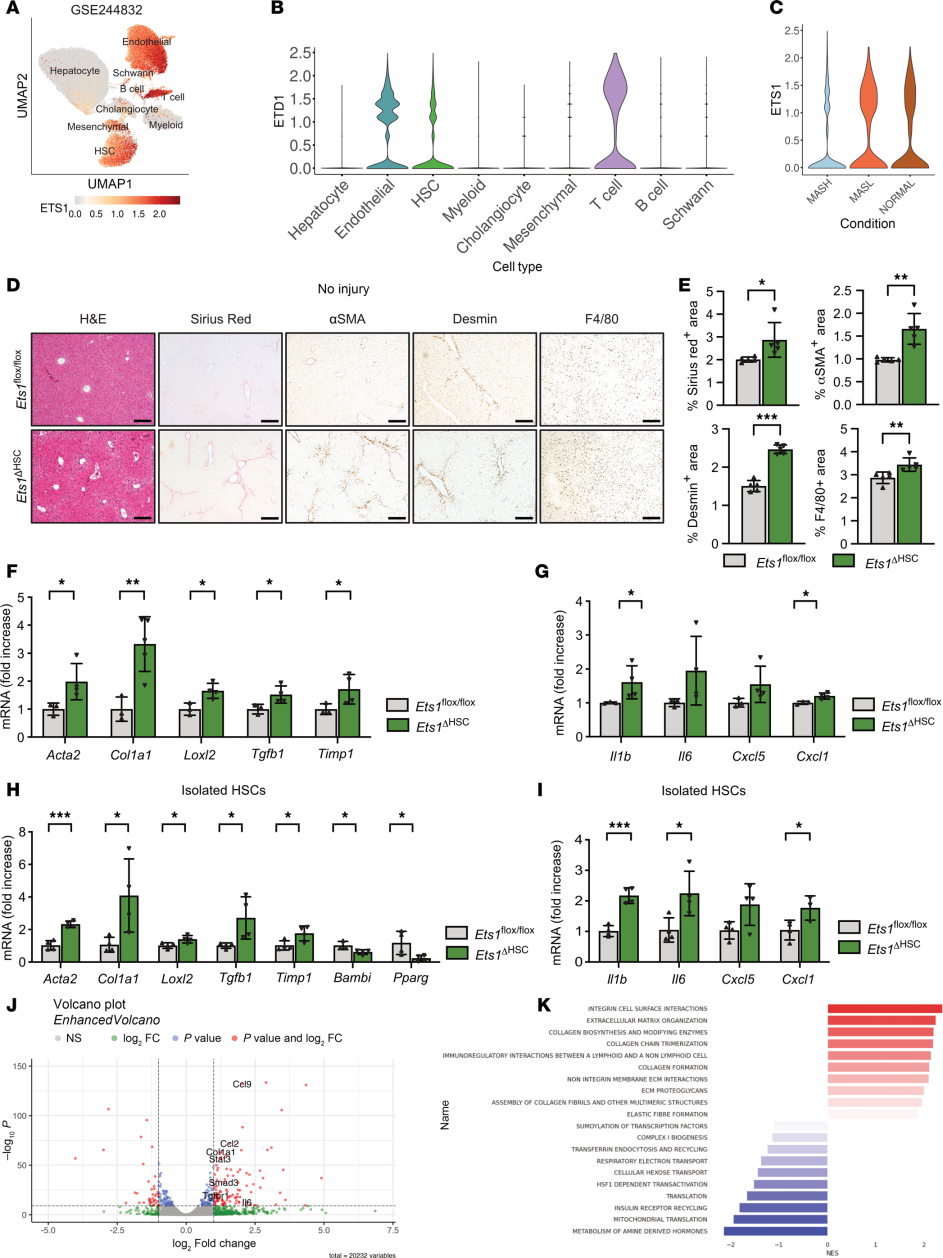
Deletion of ETS1 in HSCs promotes liver fibrosis in naive *Ets1*^ΔHSC^ mice. (**A**) UMAP plot of snRNA-Seq data from liver cells of all donors, showing identified cell types and a red color-coded UMAP for ETS1 expression. (**B**) Violin plot showing the expression of ETS1 in each subset of liver cells. (**C**) Violin plot showing the expression of ETS1 in human liver samples from human normal (*n* = 5), MASL (*n* = 4), MASH (*n* = 9) groups. (**D**) Livers from *Ets1^fl/fl^* mice and *Ets1*^ΔHSC^ mice (*n* = 3–5 per group) were stained for H&E, Sirius red, αSMA, desmin, and F4/80 (scale bar: 200 μm). (**E**) Positive area was calculated as a percentage. (**F**) Expression of fibrogenic genes and (**G**) inflammatory genes in the liver tissues were analyzed by using qRT-PCR. (**H**) Fibrogenic genes and (**I**) inflammatory genes in activated HSCs isolated from *Ets1^fl/fl^* mice and *Ets1*^ΔHSC^ mice were measured by qRT-PCR. (**J** and **K**) Bulk RNA-Seq of mouse HSCs infected with nontargeting shRNA (control) or Ets1 targeting shRNA (Ets1 knockdown). (**J**) Volcano plot of control versus Ets1 targeted HSCs. Differentially expressed genes (log_2_FC > 1.5, *P* < 10^–10^) are shown in red. (**K**) Reactome pathway enrichment analysis of control versus Ets1-knockdown HSCs. Data are expressed as the mean ± SD; *P < 0.05, **P < 0.01, and ***P < 0.001, unpaired Student’s *t* test.

ETS1 is a lineage-specific TF implicated in the maintenance of the HSC phenotype (6). Analysis of single-nucleus RNA sequencing (snRNA-Seq) of human livers (7) revealed that ETS1 is mainly expressed in HSCs, endothelial cells, and T cells (Figure 1, A and B). Expression of ETS1 was compared in human HSCs from normal liver (*n* = 5), metabolic dysfunction–associated steatotic liver (MASL) (*n* = 4), and MASH (*n* = 9) livers. Notably, HSCs from normal livers and MASL exhibited higher expression of ETS1 compared with HSCs from MASH livers with stage 2–4 fibrosis (Figure 1C), suggesting that activation of HSCs is associated with downregulation of ETS1.

### Deletion of Ets1 in HSCs promotes fibrogenesis in livers of naive Ets1^ΔHSC^ mice.

To study ETS1 function in HSCs, HSC-specific Ets1-KO (*Ets1*^ΔHSC^) mice were generated by crossing Lrat^Cre^ mice and *Ets1*-floxed mice (C57BL/6, males, *n* = 3–5/group). *Ets1*^ΔHSC^ mice grew and developed normally; however, at the age of 10 weeks on a chow diet, *Ets1*^ΔHSC^ mice exhibited signs of mild liver fibrosis and inflammation. Histological analysis revealed that the positive areas of Sirius red, αSMA, desmin, and F4/80 staining were increased in the livers of naive *Ets1*^ΔHSC^ mice compared with WT *Ets1*-floxed littermates (Figure 1, D and E). Expression of fibrogenic markers (*Acta2*, *Tgfb1*, *Col1a1*, *Loxl2*, and *Timp1*) and proinflammatory genes (*Il1b*, *Il6*, *Cxcl5*, and *Cxcl1*) was increased in the livers of *Ets1*^ΔHSC^ mice (Figure 1, F and G).

Primary HSCs were isolated from the livers of WT and *Ets1*^ΔHSC^ mice and analyzed by qRT-PCR. A high efficiency of Cre-lox recombination was achieved in Ets1-deficient HSCs compared with WT HSCs (Supplemental Figure 1A; supplemental material available online with this article; https://doi.org/10.1172/jci.insight.195242DS1). *Ets1*-deficient HSCs significantly upregulated expression of fibrogenic genes (*Acta2*, *Tgfb1*, *Col1a1*, *Loxl2*, and *Timp1*) (Figure 1H, compared with WT HSCs) but downregulated the expression of quiescence-associated genes such as *Bambi* and *Pparg* (Figure 1H). Moreover, the expression of proinflammatory genes *Il1b*, *Il6*, *Cxcl1*, and *Cxcl5* was slightly induced in *Ets1*^ΔHSC^ HSCs (Figure 1I), suggesting that deletion of *Ets1* promotes fibrogenic HSC activation in the livers of uninjured mice.

### Knockdown of Ets1 facilitates activation of primary mouse HSCs.

The gene expression profiles of mouse HSCs, in which *Ets1* was knocked down using lentiviral infection, were compared with those of control (nontargeting) HSCs using RNA-Seq (Supplemental Table 1) (6). Knockdown of *Ets1* significantly increased the expression of fibrogenic genes (*Smad3*, *Col1a1*, and *Tgfbr1*) and proinflammatory genes (*Ccl2*, *Ccl9*, and *Il6*) (Figure 1J) in culture-activated HSCs. Pathway enrichment analysis revealed a strong enrichment of genes associated with extracellular matrix proteins, including the collagen biosynthesis pathway, collagen chain trimerization, and crosslinking of collagen fibrils in *Ets1*-knockdown HSCs (Figure 1K). Similarly, upregulation of genes involved in the collagen production pathway, TGF-β receptor signaling, and IL-6 family signaling was detected in *Ets1*-knockdown HSCs by gene set enrichment analysis (GSEA) (Supplemental Figure 1, B–E), indicating that downregulation of *Ets1* drives HSC activation.

### The development and regression of liver fibrosis are impaired in CCl_4_-injured Ets1^ΔHSC^ mice.

**Figure 2:**
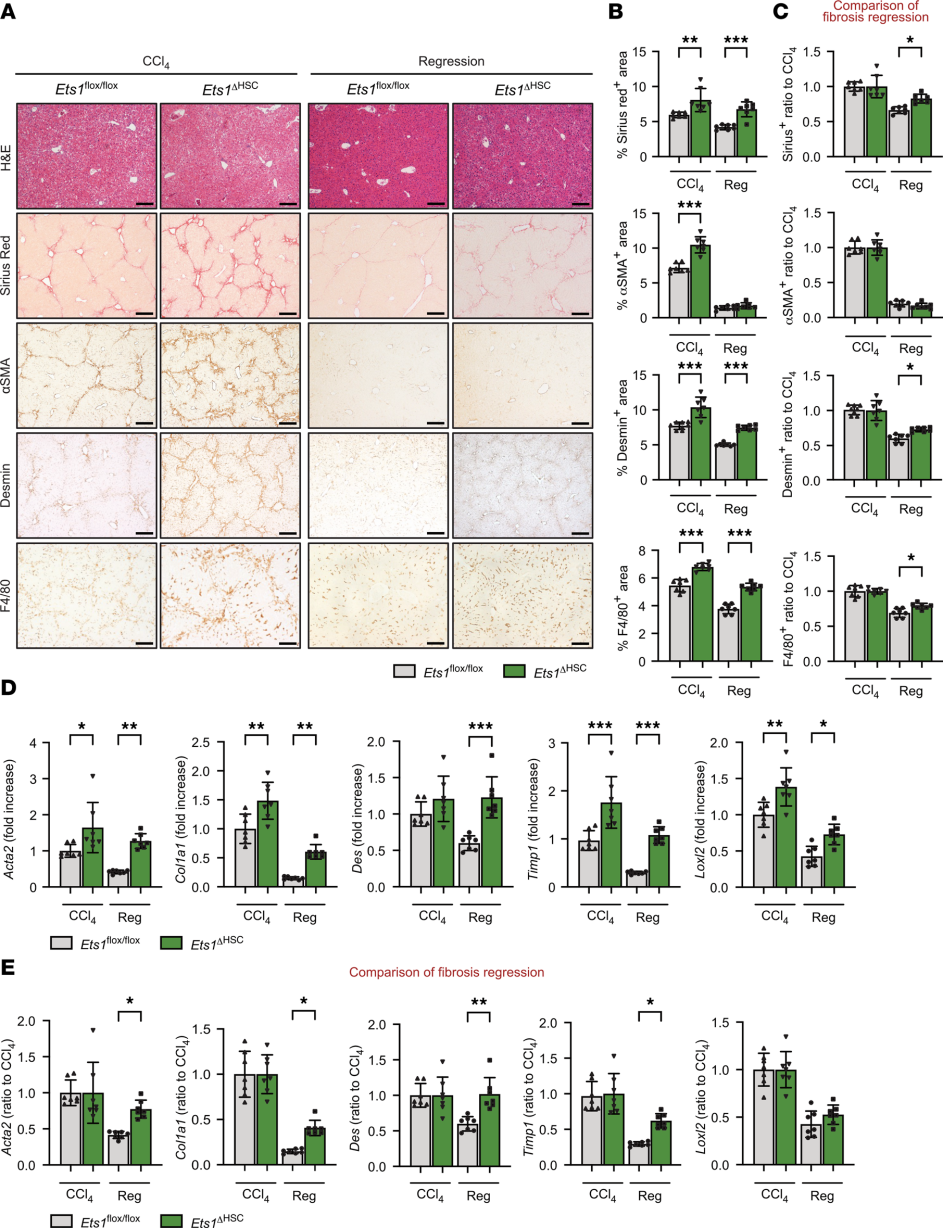
Deletion of ETS1 in HSCs exacerbates CCl_4_-induced liver fibrosis and prevents fibrosis resolution in *Ets1^ΔHSC^* mice. *Ets1^fl/fl^* mice and *Ets1*^ΔHSC^ mice (*n* = 7 per group) were administered with either vehicle (corn oil) or carbon tetrachloride (CCl_4_) for 6 weeks, followed by cessation for 2 weeks after the last CCl_4_ administration. (**A**) Livers from *Ets1^fl/fl^* mice and *Ets1*^ΔHSC^ mice were stained for H&E, Sirius red, αSMA, desmin, and F4/80 (scale bar: 200 μm), and (**B**) staining-positive area was calculated as a percentage. (**C**) The rate of fibrosis resolution was calculated by setting the positive area (%) of the group administered CCl_4_ for 6 weeks to 1 (baseline). (**D**) Fibrogenic mRNA expression in the liver tissues was analyzed by qRT-PCR. (**E**) The rate of fibrosis resolution was calculated by setting the mRNA expression of the 6-week CCl_4_-injured group to 1 (baseline). Data are expressed as the mean ± SD; *P < 0.05, **P < 0.01, and ***P < 0.001, 1-way ANOVA followed by Tukey’s test.

To explore the role of ETS1 in the development and regression of liver fibrosis, WT and *Ets1*^ΔHSC^ mice (*n* = 6 per group) were subjected to CCl_4_ liver injury for 6 weeks, followed by cessation of liver injury for 2 weeks (*n* = 6 per group). CCl_4_-injured *Ets1*^ΔHSC^ mice developed approximately 20% more liver fibrosis than WT littermates treated under the same conditions, as shown by the increased area of Sirius red, αSMA, desmin, and F4/80 staining (Figure 2, A and B) and upregulation of *Acta2*, *Col1a1*, *desmin*, *Loxl2*, and *Timp1* mRNA expression. To determine whether the upregulation of desmin expression was associated with increased HSC proliferation, livers of CCl_4_-injured WT and *Ets1*^ΔHSC^ mice were stained with anti-Ki67 antibody. Ki67 mRNA was increased in primary HSCs isolated from livers of CCl_4_-injured *Ets1*^ΔHSC^ mice (Supplemental Figure 2A). Consistently, the number of Ki67^+^ cells was significantly elevated in *Ets1*^ΔHSC^ livers compared with WT mice (Supplemental Figure 2B), indicating that loss of ETS1 directly promotes HSC proliferation.

To evaluate fibrosis resolution, resorption of fibrous scars and myofibroblast inactivation were compared in recovering mice versus CCl_4_-injured mice (14). The regression rate was calculated as the ratio of fibrogenic gene expression in recovering mice relative to the corresponding CCl_4_-injured mice (considered as 1.0 for each genotype). As shown in Figure 2C, Sirius red–positive and desmin-positive areas were increased approximately 15%–20% in livers of *Ets1*^ΔHSC^ mice recovering from CCl_4_ (vs. WT mice). To further validate this finding, quantitative digital analysis of Sirius red–stained liver sections was performed using QuPath software, which automatically detected and color-coded collagen-positive regions (red overlay) and calculated the percentage of Sirius red–positive area (Supplemental Figure 2C). We confirmed that *Ets1*^ΔHSC^ mice exhibited approximately 15%–20% higher Sirius red–positive area compared with WT mice during the regression phase, indicating impaired fibrosis resolution. Expression of *Acta2* and *Col1a1*, *desmin*, and *Timp1* mRNA remained upregulated (>35%) in livers of recovering *Ets1*^ΔHSC^ mice (Figure 2, D and E). Serum alanine aminotransferase (ALT) levels were significantly elevated in CCl_4_-injured *Ets1*^ΔHSC^ mice compared with WT mice and remained higher in *Ets1*^ΔHSC^ mice during the regression phase (Supplemental Figure 2D), indicating that liver injury is exacerbated in CCl_4_-injured *Ets1*^ΔHSC^ mice while recovery is delayed. In accordance with this finding, expression of matrix-degrading proteases (Mmp2, Mmp9, and Mmp13) was markedly reduced in livers of *Ets1*^ΔHSC^ mice and specifically in isolated *Ets1*-deficient HSCs (Supplemental Figure 2, E and F), suggesting that Ets1 deficiency in HSCs impairs extracellular matrix remodeling required for scar resolution. Taken together, these results demonstrate that the genetic deletion of *Ets1* in HSCs aggravates CCl_4_-induced liver fibrosis and prevents the regression of CCl_4_-induced liver fibrosis.

### Genetic deletion of Ets2 in HSCs does not cause liver injury in naive Ets2^ΔHSC^ mice but accelerates liver fibrosis in CCl_4_-injured Ets2^ΔHSC^ mice.

**Figure 3:**
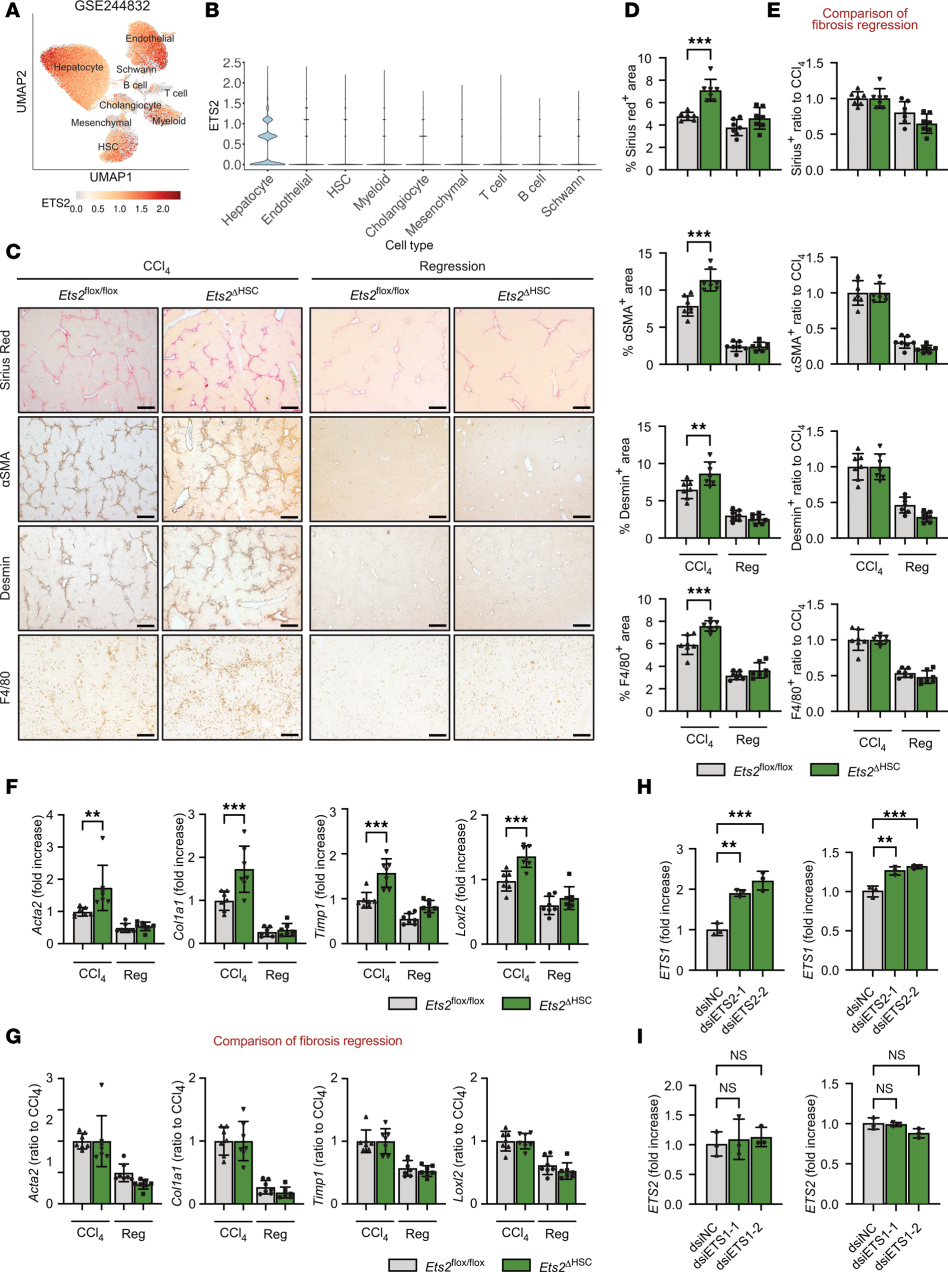
The development of liver fibrosis is increased in CCl_4_-injured *Ets2^ΔHSC^* mice. (**A**) UMAP plot of snRNA-Seq data from liver cells of all donors, showing identified cell types and a red color-coded UMAP for ETS2 expression. (**B**) Violin plot showing the expression of ETS2 in each subset of liver cells. (**C**–**G**) *Ets2^fl/fl^* mice and *Ets2*^ΔHSC^ mice (*n* = 7 per group) were administered with either vehicle (corn oil) or CCl_4_ for 6 weeks, followed by cessation for 2 weeks after the last CCl_4_ administration. (**C**) Livers from *Ets2^fl/fl^* mice and *Ets2*^ΔHSC^ mice were stained for Sirius red, αSMA, desmin, and F4/80 (scale bar: 200 μm), and (**D**) staining-positive area was calculated as a percentage. (**E**) The rate of fibrosis resolution was calculated by setting the positive area (%) of the 6-week CCl_4_-injured group to 1 (baseline). (**F**) Fibrogenic mRNA expression in the liver tissues was analyzed by qRT-PCR. (**G**) The rate of fibrosis resolution was calculated by setting the mRNA expression of the 6-week CCl_4_-injured group to 1 (baseline). (**H** and **I**) Human HSCs from two normal livers were transfected with ETS1-targeting or ETS2-targeting dsiRNA for 48 hours. (**H**) mRNA expression of ETS1 was measured in HSCs transfected with ETS2-targeting dsiRNA. (**I**) mRNA expression of ETS2 was measured in HSCs transfected with ETS1-targeting dsiRNA. Data are expressed as the mean ± SD; **P < 0.01, and ***P < 0.001, 1-way ANOVA followed by Tukey’s test.

ETS1 and ETS2 TFs share high homology. ETS2 has also been implicated in the pathogenesis of liver fibrosis (15). Unlike ETS1, ETS2 is predominantly expressed in hepatocytes, and to a lesser extent in HSCs and endothelial cells, as shown by snRNA-Seq analysis of human livers (Figure 3, A and B) (7). To investigate the role of ETS2 in HSCs, we generated *Ets2*^ΔHSC^ mice (by crossing Lrat^Cre^ mice with *Ets2*-floxed mice), in which the Ets2 gene was genetically deleted in HSCs (*n* = 3–5 per group). The loss of Ets2 expression in HSCs was validated by qRT-PCR and confirmed strong downregulation of Ets2 expression in isolated *Ets2*^ΔHSC^ HSCs compared with WT HSCs (Supplemental Figure 3A). In contrast to chow-fed naive *Ets1*^ΔHSC^ mice, deletion of *Ets2* in HSCs did not activate profibrogenic responses in naive *Ets2*^ΔHSC^ mice under physiological conditions, as shown by similar expression levels of Sirius red, αSMA, desmin, and F4/80 (Supplemental Figure 3, B and C), as well as *Acta2*, *Col1a1*, *Col1a2*, *Loxl2*, *Timp1*, and *Serpine1* in the livers of uninjured WT and *Ets2*^ΔHSC^ mice (Supplemental Figure 3D).

Deletion of *Ets2* in HSCs exacerbated liver fibrosis in CCl_4_-injured *Ets2*^ΔHSC^ mice. CCl_4_-injured *Ets2*^ΔHSC^ mice developed approximately 15%–20% more fibrosis than *Ets2*-floxed mice (*n* = 7 per group), as shown by increased expression of Sirius red, αSMA, desmin (Figure 3, C–E), and fibrogenic genes (*Acta2*, *Col1a1*, *Loxl2*, and *Timp1*) (Figure 3, F and G). However, deletion of *Ets2* in HSCs had no effect on fibrosis resolution in *Ets2*^ΔHSC^ mice recovering from CCl_4_ injury (vs. WT mice), suggesting that despite similarities, ETS1 and ETS2 mediate specific functions. In support of this finding, Ets1 expression was upregulated in ETS2-deficient HSCs compared with WT HSCs, indicating that increased levels of Ets1 may compensate for the loss of Ets2 function in naive *Ets2*^ΔHSC^ mice (Supplemental Figure 3E).

### ETS1 is a predominant isoform in human and mouse HSCs.

To test this hypothesis, ETS2 was knocked down in primary human quiescent HSCs isolated from human livers. ETS2 knockdown in human HSCs resulted in a significant increase of ETS1 expression (Figure 3H). In contrast, knockdown of ETS1 did not affect expression levels of ETS2 (Figure 3I). These findings indicate that ETS1 is a predominant isoform in HSCs, and ETS2 cannot compensate for the loss of ETS1.

To further assess functional differences and similarities between *Ets1* and *Ets2* in mouse HSCs, the gene expression profiles of mouse shRNA-*Ets1*–, shRNA-*Ets2*–, or shRNA-*Ets1/2*–knockdown HSCs were compared with shRNA-control HSCs using RNA-Seq (Supplemental Figure 4A). Knockdown of Ets1 in HSCs yielded a striking phenotype in Ets1-knockdown HSCs compared with control HSCs (9,830 genes were significantly changed out of a total of 20,231 genes). In contrast, Ets2-knockdown HSCs mainly resembled control HSCs, but they uniquely upregulated genes that were not induced in Ets1-knockdown HSCs. Simultaneous knockdown of both Ets1 and Ets2 in HSCs resulted in a third phenotype, in which the effect of Ets1 knockdown was partially reversed.

Despite the differences in the gene expression profiles, out of 100 most significantly changed pathways (selected for each group of HSCs from significantly upregulated and downregulated genes), approximately half of the top pathways overlapped between the shRNA-*Ets1*–, shRNA-*Ets2*–, or shRNA-*Ets1/2*–knockdown groups (Supplemental Figure 4B), supporting the notion that Ets1 and Ets2 exhibit some functional redundancy. In Ets1-knockdown HSCs, 33 pathways were uniquely upregulated, including those related to inflammatory responses, such as cytokine and interleukin signaling, cell-cell communications, and TLR cascades (Supplemental Figure 4, B and C). In the Ets2 knockdown group, 19 pathways were upregulated exclusively and were related to lipoprotein metabolism (Supplemental Figure 4, B and D). These findings indicate that ETS1 regulates inflammatory responses in quiescent HSCs, which may contribute to the phenotypic differences observed between shRNA-*Ets1*– and shRNA-*Ets2*–knockdown HSCs, and between *Ets1*^ΔHSC^ and *Ets2*^ΔHSC^ mice. Overall, Ets1 is highly expressed in HSCs and plays a more significant role in liver fibrosis than Ets2, since it can compensate for the loss of Ets2 functions.

### ETS1 knockdown increases responses of human HSCs to TGF-β1 stimulation.

**Figure 4:**
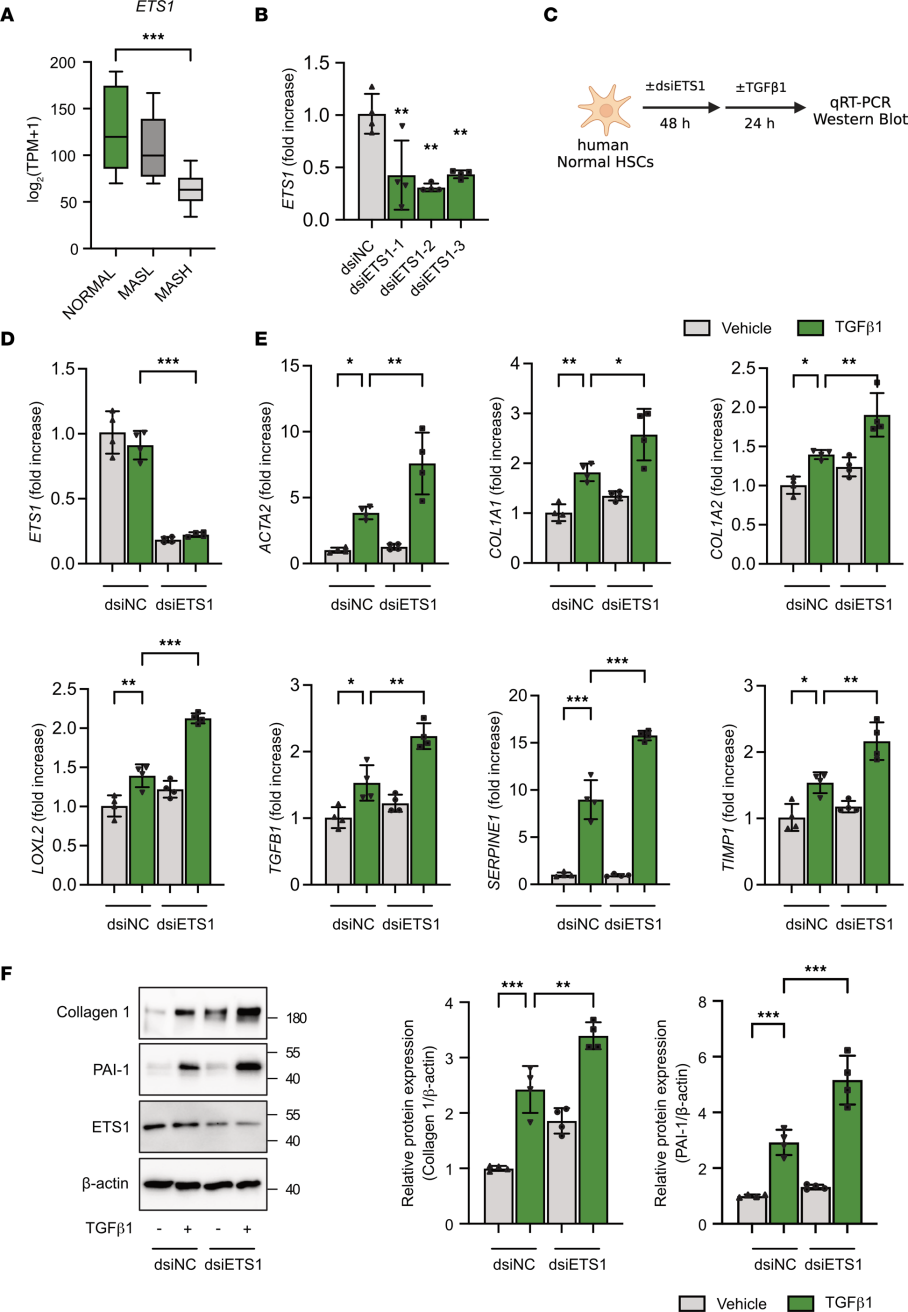
HSC-specific knockdown of ETS1 facilitates activation of TGF-β1–stimulated human HSCs. (**A**) ETS1 expression was evaluated in an RNA-Seq dataset of isolated human HSCs (normal *n* = 8, MASL *n* = 8, and MASH *n* = 12). (**B**) Human HSCs from a normal donor were transfected with 3 different ETS1-targeting dsiRNA or dsiNC (dsi-negative control) for 48 hours, and the efficiency of gene knockdown was measured by qRT-PCR. (**C**) Experimental timeline; human HSCs were transfected with ETS1-targeting dsiRNA or dsiNC for 48 hours and treated with recombinant human TGF-β1 protein (5 ng/mL) for 24 hours. (**D**) mRNA expression of *ETS1* after ETS1 knockdown in HSCs. (**E**) mRNA and (**F**) protein expression of fibrogenic markers were measured in ETS1-targeting dsiRNA-transfected (vs. dsiNC) HSCs with or without TGF-β1. Data are expressed as the mean ± SD; *P < 0.05, **P < 0.01, and ***P < 0.001, 1-way ANOVA followed by Tukey’s test.

To translate our findings in mice to humans, expression of ETS1 was analyzed in human primary HSCs isolated from normal (*n* = 8), MASL (*n* = 8), and MASH (*n* = 12) human livers. Similar to snRNA-Seq findings (Figure 1C), RNA-Seq analysis of isolated human HSCs revealed that expression of ETS1 was downregulated in MASH HSCs compared with normal livers, confirming that activation of HSCs is associated with suppression of ETS1 function (Figure 4A).

Next, primary human HSCs expressing high levels of ETS1 were isolated from normal livers and transfected with ETS1-targeting or control dsiRNA to knock down ETS1 (Supplemental Table 2). Three dsiRNA-hairpins were tested. The hairpin with highest knockdown efficiency in dsiRNA-ETS1 HSCs (~70% vs. dsiRNA-control HSCs) was used (Figure 4B). Responses of ETS1 knockdown and control HSCs to TGF-β1 were tested. Knockdown of ETS1 facilitated TGF-β1–mediated HSC activation and significantly increased expression of *ACTA2*, *COL1A1*, *COL1A2*, *LOXL2*, *TGFB1*, *SERPINE1*, and *TIMP1* mRNA (vs. control HSCs; Figure 4, C–E). Consistently, ETS1 knockdown also increased TGF-β1–induced SMAD2 phosphorylation (Supplemental Figure 5A), confirming that the amplified fibrogenic responses in ETS1-knockdown HSCs are mediated through activation of canonical TGF-β/SMAD signaling. Collagen type 1 and PAI-1 proteins were also significantly increased in ETS1-knockdown HSCs compared with controls (Figure 4F), indicating that ETS1 plays an important role in the maintenance of a quiescent HSC phenotype.

### Knockdown of ETS1 in HSCs exacerbates MASH fibrosis in 3D human liver spheroids.

**Figure 5:**
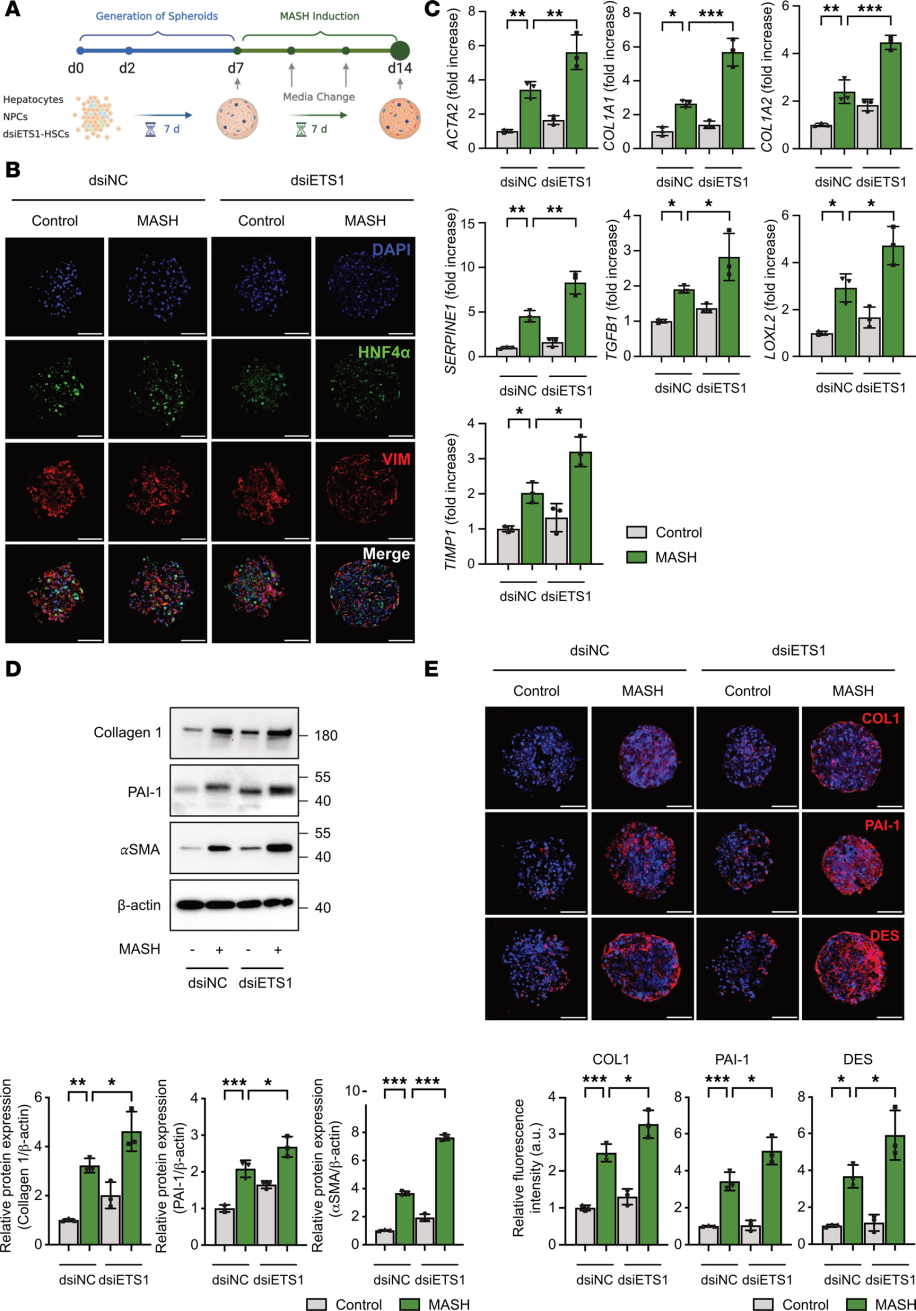
Knockdown of ETS1 in HSCs accelerates liver fibrosis in human liver spheroids with MASH. (**A**) Experimental timeline; human HSCs were transfected with ETS1-targeting dsiRNA or dsiNC (dsi-negative control) for 48 hours, and human liver spheroids were generated by coculturing different liver cells for 7 days, followed by stimulation with MASH cocktail for an additional 7 days. (**B**) Immunofluorescence images of human control and MASH liver spheroids containing ETS1-knockdown HSCs were stained for hepatocyte nuclear factor 4α (HNF-4α), vimentin (VIM), and DAPI (scale bar: 100 μm). Fibrogenic markers in control and MASH liver spheroids were assessed using (**C**) qRT-PCR and (**D**) Western blot analysis. (**E**) Control and MASH liver spheroids containing ETS1-knockdown HSCs were stained for collagen 1, PAI-1, and desmin (scale bar: 100 μm). Data are expressed as the mean ± SD; *P < 0.05, **P < 0.01, and ***P < 0.001, 1-way ANOVA followed by Tukey’s test.

The role of ETS1 in activation of human HSCs was evaluated using 3D human liver spheroids with MASH (16). HSCs were transfected with ETS1-targeting or control dsiRNA before spheroid formation and then used for spheroid generation. Human liver spheroids were generated by coculturing all liver cell types — hepatocytes (3 × 10^5^), nonparenchymal cells (1.5 × 10^5^), and HSCs (0.8 × 10^5^) — at physiological ratios in the presence of MASH cocktail (160 μM palmitate, 160 μM oleate, 10 mM fructose, 5.5 mM glucose, 10 μg/mL LPS, 1 ng/mL TGF-β1) (Figure 5A). Spheroids (*n* = 3 per group) were harvested and analyzed. Hepatocytes and HSCs were visualized by immunostaining for hepatocyte nuclear factor 4α (HNF-4α) and vimentin, respectively (Figure 5B). As expected, exposure to the MASH cocktail increased expression of fibrogenic markers (e.g., *ACTA2*, *COL1A1*, *COL1A2*, *SERPINE1*, *TGFB1*, *LOXL2*, and *TIMP1* mRNA and collagen type I, αSMA, and PAI-1 proteins) in human liver spheroids (Figure 5C) compared with spheroids cultured in growth medium (Figure 5, D and E). When MASH spheroids were compared, knockdown of ETS1 in HSCs resulted in a significant increase of approximately 30% of fibrogenic gene expression in human liver spheroids with MASH (Figure 5, C–E). Remarkably, upregulation of collagen type 1, PAI-1, αSMA, and desmin proteins was observed in human liver spheroids containing ETS1-knockdown HSCs even prior to injury and was further increased (~4 fold) in response to MASH (Figure 5, D and E).

### Knockdown of ETS1 in human HSCs decreases regression of MASH-induced liver fibrosis in human liver spheroids.

**Figure 6:**
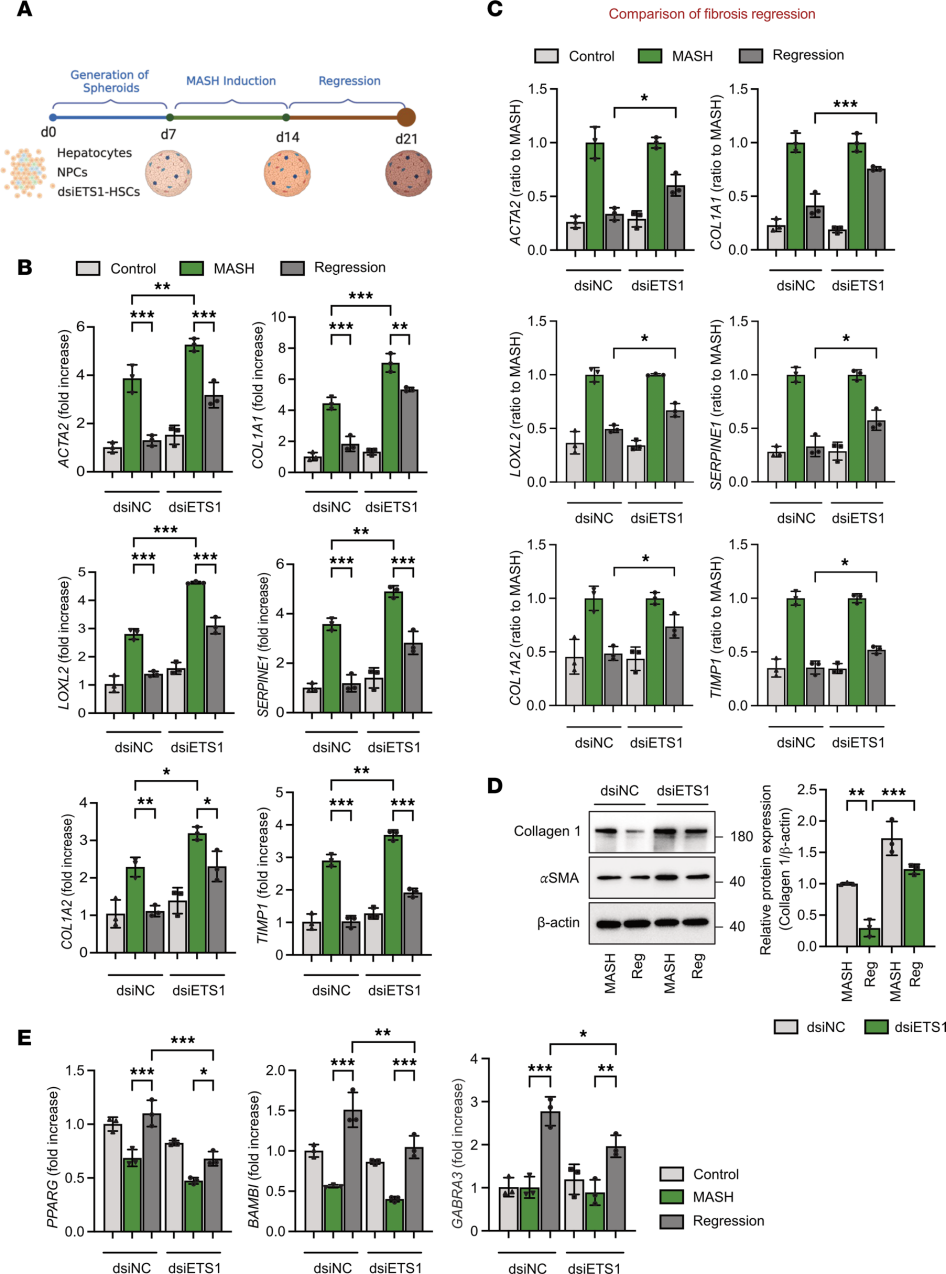
Knockdown of ETS1 in HSCs attenuates the regression of MASH-induced liver fibrosis in human liver spheroids. (**A**) Experimental timeline; human HSCs were transfected with ETS1-targeting dsiRNA or dsiNC (dsi-negative control) for 48 hours, and human liver spheroids were generated by coculturing different liver cells for 7 days. After formation, metabolic liver fibrosis was induced by stimulation with MASH cocktail for an additional 7 days, followed by removal of metabolic injury by replacing MASH cocktail with growth medium. (**B**–**E**) Fibrogenic markers in liver spheroids were assessed using (**B**) qRT-PCR or (**D**) Western blot analysis, and (**C**) the rate of fibrosis resolution was quantified by setting the mRNA expression of the MASH-induced group to 1 (baseline). (**E**) mRNA expression of inactivation-associated HSC markers was measured. Data are expressed as the mean ± SD; *P < 0.05, **P < 0.01, and ***P < 0.001, 1-way ANOVA followed by Tukey’s test.

Regression of liver fibrosis was achieved by transferring MASH spheroids into growth medium (16). Culturing MASH liver spheroids in growth medium caused HSC inactivation, which was characterized by downregulation of fibrogenic gene expression and reexpression of some quiescence-associated genes (Figure 6A) (4, 17). Consistent with in vivo findings, MASH liver spheroids containing ETS1-targeted HSCs exhibited an approximately 30% lower rate of fibrosis regression compared with control spheroids, as shown by higher expression of *ACTA2*, *COL1A1*, *LOXL2*, *SERPINE1*, *COL1A2*, and *TIMP1* (Figure 6, B and C). We also observed persistence of collagen type 1 and αSMA protein expression in human liver spheroids containing *ETS1*-targeted HSCs during regression of MASH fibrosis (Figure 6D). Moreover, expression of inactivation markers PPARG, BAMBI, and GABRA3 was significantly lower in ETS1 knockdown liver spheroids compared with control spheroids (Figure 6E). These results indicate that HSC-specific knockdown of ETS1 suppresses the inactivation of human HSCs and prevents regression of liver fibrosis in MASH spheroids.

### CRTC2 is a potential ETS1 target gene in human HSCs.

**Figure 7:**
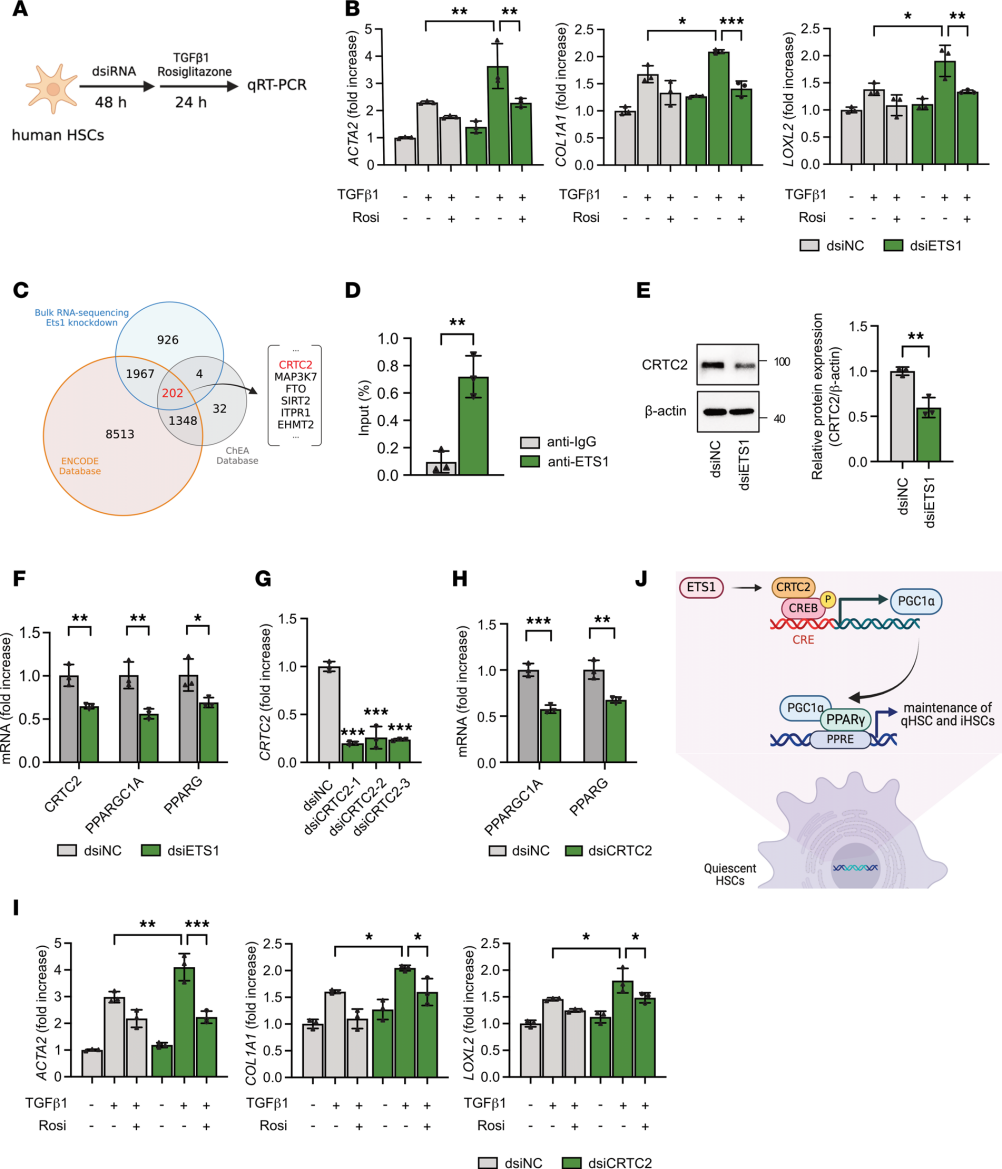
ETS1 regulates PPARγ via the CRTC2 pathway in human HSCs. (**A**) Schematic illustration of dsiRNA transfection and rosiglitazone treatment. Human HSCs were transfected with dsiRNA for 48 hours and treated with recombinant human TGF-β1 protein (5 ng/mL) with or without rosiglitazone (20 μM or vehicle) for 24 hours. (**B**) The expression of fibrogenic genes was measured in ETS1-targeting dsiRNA-transfected HSCs after rosiglitazone treatment by qRT-PCR. (**C**) ETS1 direct target genes were overlapping with the genes downregulated in ETS1 knockdown HSCs (vs. HSCs infected with nontargeting shRNA) in our RNA-Seq dataset. ETS1 target genes were identified from the ENCODE and ChEA databases. (**D**) ETS1 locus-specific ChIP-qPCR analysis was performed using human HSCs. (**E** and **F**) Human HSCs were transfected with ETS1-targeting dsiRNA or dsiNC (dsi-negative control). (**E**) CRTC2 and (**F**) CRTC2 downstream genes were measured using Western blot analysis or qRT-PCR. (**G**) Human HSCs were transfected with 3 different CRTC2-targeting dsiRNA or dsiNC (dsi-negative control) for 48 hours, and the efficiency of gene knockdown was measured by qRT-PCR. (**H**) mRNA expression of PPARGC1A and PPARG in CRTC2-targeting dsiRNA-transfected HSCs. (**I**) mRNA expression of fibrogenic markers was measured in dsi*CRTC2*-transfected HSCs. (**J**) Schematic illustration of the underlying mechanism by which ETS1 regulates the HSC phenotype through CRTC2. Data are expressed as the mean ± SD; *P < 0.05, **P < 0.01, and ***P < 0.001, 1-way ANOVA followed by Tukey’s test.

PPARγ plays a critical role in the maintenance of the quiescent HSC phenotype (6), and PPARγ was identified as an ETS1 target gene (18). In support of this finding, ETS1 knockdown using dsiRNA in human HSCs abrogated PPARγ-mediated effects in TGF-β1–stimulated HSCs, leading to exacerbation of fibrogenic responses, and treatment with the PPARγ agonist rosiglitazone (20 μM) effectively restored PPARγ signaling in ETS1-knockdown HSCs (Figure 7, A and B). Still, the mechanism by which ETS1 regulates PPARγ is unknown. We used the ENCODE (Encyclopedia of DNA Elements) (19) and ChEA (ChIP Enrichment Analysis) (20) databases, which are designed to identify the target genes of TFs based on their DNA binding site profiles, to investigate the ETS1/PPARγ axis. Using this approach, potential ETS1 targets were selected from the list of genes downregulated in mouse HSCs with shRNA-mediated Ets1 knockdown in the RNA-Seq dataset (Figure 7C). We identified 202 potential ETS1 target genes, including CRTC2, which has been implicated in PPARγ activation (21). Furthermore, FIMO-based motif enrichment analysis of single nucleus ATAC-Seq data of human livers (7) revealed that ETS1 binds to the regulatory element of the CRTC2 gene locus, suggesting ETS1 as a potential regulator of CRTC2 transcription (Supplemental Figure 6, A and B). To test this hypothesis, ChIP-qPCR was performed in human HSCs using an anti-ETS1 antibody (targeting the promoter region of the CRTC2 gene where putative ETS1 binding motifs were identified by in silico analysis). ETS1 binding was significantly enriched at the CRTC2 promoter region, whereas no enrichment was observed in the IgG control (Figure 7D). These results provide direct mechanistic evidence that ETS1 binds to the CRTC2 locus and positively regulates CRTC2 transcription, supporting the notion that ETS1 regulates the CRTC2/PPARγ axis to maintain the quiescent HSC phenotype.

### ETS1 regulates the quiescent HSC phenotype via activation of the CRTC2/PGC1α/PPARγ pathway in human HSCs.

CRTC2 (TORC2) is a transcriptional coactivator of CREB TF that also regulates hepatic gluconeogenesis-related genes, including transcription of PGC1α, encoded by the PPARGC1A gene. PGC1α can directly interact with PPARγ (22). We hypothesized that ETS1 modulates PPARγ responses through activation of the CRTC2/PGC1α signaling pathway in HSCs. In support of this hypothesis, dsiRNA-based ETS1 knockdown in human HSCs significantly suppressed CRTC2 expression, leading to downregulation of PPARGC1A and PPARG (Figure 7, E and F). Similarly, expression of PPARGC1A and PPARG was reduced in CRTC2-knockdown HSCs (Figure 7, G and H). To further validate the role of ETS1 in the regulation of the CRTC2/PGC1α/PPARγ pathway, expression of CRTC2, PGC1A, and PPARG was measured in mouse and human HSCs. HSCs isolated from uninjured *Ets1*^ΔHSC^ mice exhibited significant downregulation of CRTC2, PGC1A, and PPARG compared with WT HSCs (Supplemental Figure 7A). Consistently, ETS1-targeted and control human HSCs were stimulated with or without TGF-β1. dsiRNA-based knockdown of ETS1 in human HSCs resulted in a significant reduction in CRTC2, PGC1A, and PPARG expression (Supplemental Figure 7B). To determine whether the effects of ETS1 and CRTC2 on HSC phenotype are mediated by PPARγ, CRTC2 was knocked down using dsiRNA in human HSCs. CRTC2-targeted and control HSCs were pretreated with or without rosiglitazone (a PPARγ agonist) and subsequently stimulated with or without TGF-β1 (Figure 7A). CRTC2 knockdown in human TGF-β1–stimulated HSCs significantly downregulated expression of PPARγ and increased fibrogenic gene expression, while rosiglitazone treatment prevented fibrogenic activation of CRTC2-targeted HSCs (Figure 7I). Overall, these results suggest that ETS1 regulates the quiescent phenotype of HSCs through a CRTC2/PGC1α/PPARγ pathway (Figure 7J).

## Discussion

Our previous research identified ETS1/2 as key lineage-determining TFs that maintain the quiescent HSC phenotype (6). Here, we demonstrate that genetic deletion of ETS1/2 in HSCs exacerbated the development of toxic liver fibrosis in mice and suppressed fibrosis resolution due to increased activation of HSCs. Similarly, knockdown of ETS1 in human liver spheroids accelerated MASH and prevented fibrosis resolution. We found that ETS1 regulates its functions via induction of CRTC2/PGC1α/PPARγ signaling in HSCs. Knockdown of CRTC2 blocked ETS1-mediated signals and prevented activation of PPARγ TF, critical for HSC quiescence and inactivation.

Regulation of HSC activation has been extensively studied. Recently, a conceptually novel paradigm of HSC regulation on multiple levels via the crosstalk between HSC lineage-determining, cluster-specific, and signal-specific (e.g., TGF-β) TFs was proposed (7). Specifically, fibrogenic activation of HSCs is driven by epigenetic/transcriptional changes in the activity of lineage-determining TFs (such as increased induction of *JUNB/AP-1* over *ETS*) that are tightly controlled by cluster-specific TFs (increased induction of aHSC-specific *RUNX1/2* over *LHX1/2*, *FXR*) and signal-specific TFs (such as FOXA1/2, FOXP1, SRF, and others). *RUNX1/2* was implicated in regulation of lineage-determining, cluster-specific, and signal-dependent TFs (23), suggesting that *RUNX1* may be an essential master regulator of HSC activation due to suppression of ETS1 and activation of JUNB/AP-1 (7).

The TFs of the Ets family, which are defined by the presence of the conserved DNA-binding ETS domain, are the master regulators of development. ETS1 is one of the most abundant TFs of this family. ETS1 is expressed in almost all liver cell types, including hepatocytes, HSCs, T cells, B cells, and NK cells (13), with particularly high expression in HSCs (24). In our study, the highest expression of ETS1 in the human liver was observed in HSCs, endothelial cells, and T cells. Despite extensive studies, the role of ETS1 in the liver is not well understood. Global deletion of *Ets1* in mice yielded a hepatic phenotype, such as suppression of hepatocyte apoptosis and improvement of diet-induced MASH, leading to reduced liver injury, inflammation, and fibrosis. Moreover, conditional knockdown of ETS1 in hepatocytes significantly ameliorated liver fibrosis and inflammation in mice with MASH (25). In contrast, HSC-specific *Ets1*-KO mice were more susceptible to TGF-β1 stimulation and CCl_4_-induced liver fibrosis, highlighting the cell type–specific role of ETS1. Similar differences in hepatocyte and HSC phenotypes were reported for cell-specific *Serpine1-*KO mice (7, 26), *Pnpla3* mutant mice (27), and *Pparg*-KO mice (6, 28). Deletion of ETS1 in hepatocytes suppressed TGF-β–induced hepatocyte apoptosis (25), and deletion of ETS1 in HSCs accelerated TGF-β–mediated HSC activation. Interestingly, the loss of ETS1 in endothelial cells impaired coronary vascular development, leading to ventricular non-compaction, increased TGF-β signaling, and excessive deposition of extracellular matrix in the trabecular layer of the heart ventricles (29). In addition to ETS1/2, other TFs such as LHX2 (30) and TCF21 (31) have been implicated in maintaining HSC quiescence, as shown in the *Lhx2*-KO model. However, in our analysis, both LHX2 and TCF21 were expressed at much lower levels than ETS1 and did not vary across disease stages. Moreover, ETS1 knockdown did not alter their expression, indicating that ETS1 does not transcriptionally regulate these genes. These findings suggest that although LHX2 and TCF21 may contribute to HSC quiescence under certain conditions, ETS1 plays a unique and nonredundant role in preventing fibrogenic activation. Our data support the notion that a group of key TFs (such as ETS1, c-JUN/AP-1, GATA, TEAD, and others) that are highly expressed in all cell types (7) are modulated on multiple levels to achieve cell-specific (and often opposing) responses to injury or stress.

Cell-specific responses to ETS1 can be explained by activation of different signaling pathways. TGF-β1/Smad2/3 drives transcription of Ets-1 in hepatocytes (25). Ets-1 can directly bind to phospho-Smad3, thereby enhancing TGF-β1–induced hepatocyte apoptosis. Therefore, Ets-1 knockdown alleviated MASH fibrosis due to reduced hepatocyte apoptosis (25). In contrast, knockdown of ETS1 in human skin fibroblasts amplified expression of TGF-β1 target genes (32). Ets1 was shown to be a potent suppressor of TGF-β1/Smad2/3 that mediates its signaling via association with the p300/CBP complexes (32). In accord, deletion of Ets1 in HSCs facilitated TGF-β1–induced HSC activation. Here, we demonstrate that ETS1 regulates the quiescent HSC phenotype via activation of the CRTC2/CREB/PGC-1α/PPARγ signal transduction cascade. CRTC2 is a direct target of ETS1. CRTC2 binds to CREB (33), and the CRTC2/CREB complex activates transcriptional coactivator PGC-1α, which drives expression of PPARγ (21, 34). Although PPARγ is a known ETS1 target gene, here we describe a mechanism of its regulation in HSCs, which to our knowledge is previously unrecognized.

Nearly 30 members of this ETS family have been identified, and they exhibit different expression patterns and mediate a wide range of physiological and pathological processes. Hence, the precise roles of each TF have not been delineated. ETS2 is another TF that exhibits high homology to ETS1 and is highly expressed in the liver, mainly in hepatocytes, HSCs, and endothelial cells. ETS1/2 are lineage-specific TFs that maintain a quiescent HSC phenotype (6). Here, we compared Ets1 and Ets2 functions in HSC-specific Ets1-KO and Ets2-KO mice. Deletion of Ets1 or Ets2 exacerbated the development of toxic liver fibrosis. Despite their structural similarities, Ets1 and Ets2 exhibited distinct properties (35). For example, naive Ets1-deficient HSCs (but not Ets2-deficient HSCs) were promptly activated. Deletion of *Ets1* had a stronger impact on liver fibrosis in CCl_4_-injured *Ets1*^ΔHSC^ mice compared with *Ets2*^ΔHSC^ mice. In addition to increased fibrosis, *Ets1-*deficient HSCs caused increased inflammation. Deletion of Ets1, but not Ets2, was critical for inactivation of HSCs during regression of liver fibrosis. Moreover, expression of Ets1 was upregulated in CCl_4_-injured *Ets2*^ΔHSC^ mice, suggesting that Ets1 can partly compensate for the loss of Ets2, whereas Ets2 could not compensate for the loss of Ets1. Our findings indicate that Ets1 is a predominant lineage-specific TF that regulates the quiescent phenotype in HSCs. In support of these findings, in vitro knockdown of ETS1 in mouse or human HSCs caused their spontaneous activation. Moreover, knockdown of ETS1 in human liver spheroids exacerbated the development of MASH liver fibrosis and prevented fibrosis resolution upon cessation of MASH-induced liver injury. Collectively, these findings suggest that modulating ETS1 activity in HSCs may provide a therapeutic approach for treatment of liver fibrosis.

In conclusion, this study demonstrates that ETS1 in HSCs plays a critical role in preventing the development of toxic liver fibrosis and promoting fibrosis resolution via activation of the ETS1/CRTC2-dependent signaling pathway.

## Methods

### Sex as a biological variable.

This study included male mice, consistent with previous studies using the same CCl_4_-induced liver fibrosis model (4). Male mice were selected because of their more extensively characterized responses in this model, allowing for improved comparability with existing data.

For human normal, MASL, and MASH livers, deidentified donor livers were obtained via Lifesharing OPO, which provided the informed consent, laboratory tests (ALT, aspartate transaminase, biochemistry, cell counts, liver biopsy, serology, and others), and patients’ history (cause of death, age, BMI, sex, and underlying diseases). Livers were graded by a pathologist using a double-blinded method and identified as normal, MASL, and MASH (7). The MASH/Clinical Research Network criteria was used to categorize livers; a score of less than 3 indicated a normal liver. Livers with steatosis (NAS < 3; steatosis present without inflammation or ballooning) were identified as MASL; livers with scores of 5 or higher (with steatosis, inflammation, and fibrosis) were diagnosed as MASH. Liver diagnoses of normal (*n* = 5), MASL (*n* = 4), and MASH (*n* = 9, fibrosis stage 2–4) were defined by a combination of patient history and liver histology. Snap-frozen livers were used for snRNA-Seq. Fresh livers were used for liver cell isolation. Isolated cells were cryopreserved and used for in vitro studies and formation of 3D human liver spheroids (16).

### Mice.

Lrat^Cre^ and *Ets1*-floxed mice (*Ets1^fl/fl^*) were gifts of Robert F. Schwabe (Columbia University, New York, New York, USA) (36) and Barbara L. Kee (University of Chicago, Chicago, Illinois, USA) (37). *Ets2*-floxed mice (*Ets2^fl/fl^*) were purchased from The Jackson Laboratory (stock 020455). HSC-specific Ets1-KO mice (*Ets1*^ΔHSC^) and HSC-specific Ets2-KO mice (*Ets2*^ΔHSC^) were generated by crossing Lrat^Cre^ mice with *Ets1^fl/fl^* or *Ets2^fl/fl^* mice. Mice were maintained under pathogen-free conditions in filter-topped cages with autoclaved food and water on a 12-hour light/12-hour dark cycle, as approved by the University of California San Diego (UCSD) IACUC (protocol S07088).

### Experimental model for liver fibrosis and regression.

Liver fibrosis was induced in *Ets1*^ΔHSC^ and *Ets2*^ΔHSC^ mice by administration of CCl_4_ (1:4 in corn oil, oral gavage, twice a week) for 6 weeks, followed by regression for liver fibrosis (2 weeks). Mice were euthanized 72 hours after the last CCl_4_ administration.

### Isolation of primary mouse HSCs.

Mouse HSCs were isolated from livers using the pronase/collagenase method and gradient centrifugation (8.2% Nycodenz, Cosmo Bio USA) (38). Freshly isolated HSCs were cultured in DMEM (11965-092, Life Technologies) supplemented with 10% FBS (100-106; Gemini Bio) and 1% antibiotics/antimycotics. HSCs were maintained at 37°C in a humidified atmosphere of 5% CO_2_.

### qRT-PCR.

RNA was extracted using PureLink RNA Mini Kit (12183018A; Life Technologies), and cDNA was prepared using High-Capacity cDNA Reverse Transcription Kit (4368813; Life Technologies). qRT-PCR was performed using a QuantStudio Real-Time PCR system (Applied Biosystems). The mRNA levels were normalized to a housekeeping gene (HPRT or 18s rRNA) by using the ΔΔCT method, and the fold-increase on the *y* axis represents the fold-change of the normalized target gene expression compared with the control. PCR primer sequences are shown in Supplemental Table 3.

### SnRNA-Seq data analysis.

Publicly available snRNA-Seq data in human livers were downloaded from National Center for Biotechnology Information (NCBI) Gene Expression Omnibus (GEO) under accession number GSE244832. Ambient RNAs were removed by using CellBender, and additional genes from background contamination were filtered as previously described (7). SnRNA-Seq data derived from 18 human liver samples were combined and clustered using Seurat R package.

### RNA-Seq data analysis.

Ets1, Ets2, or Ets1/2 were knocked down with shRNAs in primary mouse HSCs and subjected to RNA-Seq. Publicly available mouse RNA-Seq data were downloaded from GEO under accession number GSE140641 (6).

### IHC.

FFPE mouse liver tissues were stained with Sirius red, anti-F4/80 (14-4801-82, 1:200; eBioscience), anti-αSMA (ab5694, 1:200; Abcam), and anti-desmin (ab15200, 1:100; Abcam) antibodies (Supplemental Table 4). Images were taken by using an Olympus microscope. Positive areas were quantified by using ImageJ (NIH).

### Western blot analysis.

Cell lysates (10–20 μg) in RIPA lysis buffer plus protease and phosphatase inhibitor cocktails (Sigma-Aldrich) were analyzed by SDS-PAGE gel and transferred to PVDF membranes. Protein bands were detected with enhanced chemiluminescence Western blotting reagent (SuperSignal West Pico PLUS Chemiluminescent substrate, 34580; Thermo Fisher Scientific). The primary antibodies and dilutions were as follows: collagen 1 (600-401-103S, 1:1,000; Rockland), PAI-1 (13801-1-AP, 1:1,000; Proteintech), ETS1 (ab124282, 1:1,000; Abcam), αSMA (ab5694, 1:2,000; Abcam), and β-actin (A5441, 1:5,000; Sigma-Aldrich) (Supplemental Table 4). Densitometric analysis was performed by using ImageJ (NIH) and normalized to β-actin.

### Isolation of human HSCs and primary human HSC culture.

Deidentified donor livers (IRB 171883XX) were obtained via Lifesharing OPO, which provided the informed consent. Human HSCs were isolated using the pronase/collagenase method from donor livers declined for transplantation (39). Primary human HSCs (passage 0–1) were maintained in DMEM supplemented with 10% FBS and 1% antibiotic/antimycotic and used for further experiments.

### dsiRNA-based gene knockdown in human HSCs.

Human HSCs were transfected with dsiRNA (Integrated DNA Technologies) using RNAiMAX lipofectamine (13778075; Thermo Fisher Scientific). Primary human HSCs (5 × 10^4^) were transfected with ETS1, ETS2, CRTC2-targeting, or negative control dsiRNA for 48 hours, followed by stimulation with or without TGF-β1 (5 ng/mL) for 24 hours. Next, 3 hairpins were tested for ETS1, ETS2, or CRTC2 knockdown efficiency, and the hairpin with the highest knockdown efficiency (>70%) was selected for further experiments (Supplemental Table 2).

### Generation of human liver spheroids.

Cryopreserved human hepatocytes (3 × 10^5^) plus nonparenchymal cells (1.5 × 10^5^) and HSCs (0.8 × 10^5^) were mixed in 96-well ultra-low attachment plates (454552, Corning) and cultured in DMEM supplemented with 10% FBS, 1% penicillin/streptomycin, 0.1 μM dexamethasone (Invivogen), and 1% insulin-transferrin-selenium (41400-045, Gibco) for 7 days; afterward, spheroids were cultured with or without MASH cocktail (160 μM palmitate, 160 μM oleate, 10 mM fructose, glucose 5.5 mM, LPS 10 μg/mL, human TGF-β1 1 ng/mL) for an additional 7 days. To achieve fibrosis regression, MASH cocktail was gradually replaced with growth medium over the period of 3 days. Spheroids were harvested and analyzed.

### Locus-specific ChIP.

ChIP (40) was performed in biological replicates using MAGnify Chromatin Immunoprecipitation System (Thermo Fisher Scientific, 492024). Human HSCs were first cross-linked with disuccinimidyl glutarate followed by 1% formaldehyde, and the reaction was quenched with 125 mM glycine. Nuclei were lysed and chromatin was sheared by sonication (20 cycles, 60 seconds, duty factor 5.0%, peak power 140 W, cycles per burst 200) to yield fragments of approximately 200–500 bp. Chromatin was immunoprecipitated using an anti-ETS1 antibody or recombinant IgG control. Primers for locus-specific ChIP-qPCR analysis were designed based on predicted ETS1 binding motifs identified by FIMO analysis.

### Statistics.

Analysis was performed using GraphPad Prism 10. Data represent mean ± SD; statistically significant differences were assessed using an unpaired 2-tailed Student’s *t* test or 1-way ANOVA analysis followed by Tukey’s multiple-comparison test. A *P* value less than 0.05 was considered statistically significant.

### Study approval.

Animal experiments were conducted under the ethical guidelines approved by the UCSD IACUC, which also approved the experiments (protocol S07088). Human liver samples were obtained from Lifesharing OPO with written informed consent, and experiments were approved under IRB 171883XX, certified by the Human Research Protections Program director and IRB chair as “no human subjects” according to the Code of Federal Regulations, title 45, part 46 and UCSD Standard Operating Policies and Procedures). 

### Data availability.

All raw data files are available upon request. Values for all data points in graphs are reported in the Supporting Data Values file.

## Author contributions

WL, XL, DD, HYK, DAB, and TK designed the research. XL generated KO mice and performed animal experiments. WL, XL, SS, KH, and HYK acquired, analyzed, and interpreted the data. SBR and CM performed bioinformatic analysis. WL, XL, SS, KH, and HYK conducted experiments, isolated human cells, and generated human liver spheroids. WL, HYK, DAB, and TK wrote and edited the manuscript. DD, DAB, and TK supervised and supported the study. The co–first authorship order reflects contributions to the study. All authors read and approved the final manuscript.

## Funding support

This work is the result of NIH funding, in whole or in part, and is subject to the NIH Public Access Policy. Through acceptance of this federal funding, the NIH has been given a right to make the work publicly available in PubMed Central.

- NIH R01DK111866, R56DK088837, DK099205, AA028550, DK101737, AA011999, DK120515, AA029019, DK091183, P42ES010337, R44DK115242 (to TK and DAB)
- NIH R01CA285997 (to DAB).
- Sanford Stem Cell Fitness and Space Medicine Center at Sanford Stem Cell Institute (UCSD) (to TK).
- National Research Foundation of Korea grant funded by the Korean government (MSIT) (RS-2025-25431473, RS-2025-16067292, and RS-2025-16067036).
- Gachon University (Korea) research fund of 2024 (GCU-202500490001).
- NIH NINDS P30NS047101 (UCSD microscopy core)

## Supplementary Material

Supplemental data

Unedited blot and gel images

Supporting data values

## Version 1 11/04/2025

In-Press Preview

## Version 2 12/22/2025

Electronic publication
