# Supplementary material for: ETS1 suppresses hepatic stellate cell activation and liver fibrosis
Source: JCI Insight. 2025 Nov 4;10(24):e195242. doi: 10.1172/jci.insight.195242 (PMC12890492; doi:10.1172/jci.insight.195242)
Supplement: Supplemental data [file jciinsight-10-195242-s084.pdf]

Supplemental Material

ETS1 suppresses Hepatic Stellate Cell activation and Liver Fibrosis

Wonseok Lee[1,2,3]†, Xiao Liu[1,2]†, Sara Brin Rosenthal[4], Charlene Miciano[5,6], Sadatsugu Sakane[1,2], Kanani Hokutan[1,2], Debanjan Dhar[1,7], Hyun Young Kim[1,2,8,9], David A. Brenner[7]*, Tatiana Kisseleva[2]*

This file includes:

Supplemental figures and figure legends: Figure S1-S7

Supplemental tables and table legends: Table S1-S4

Supplemental figures and figure legends

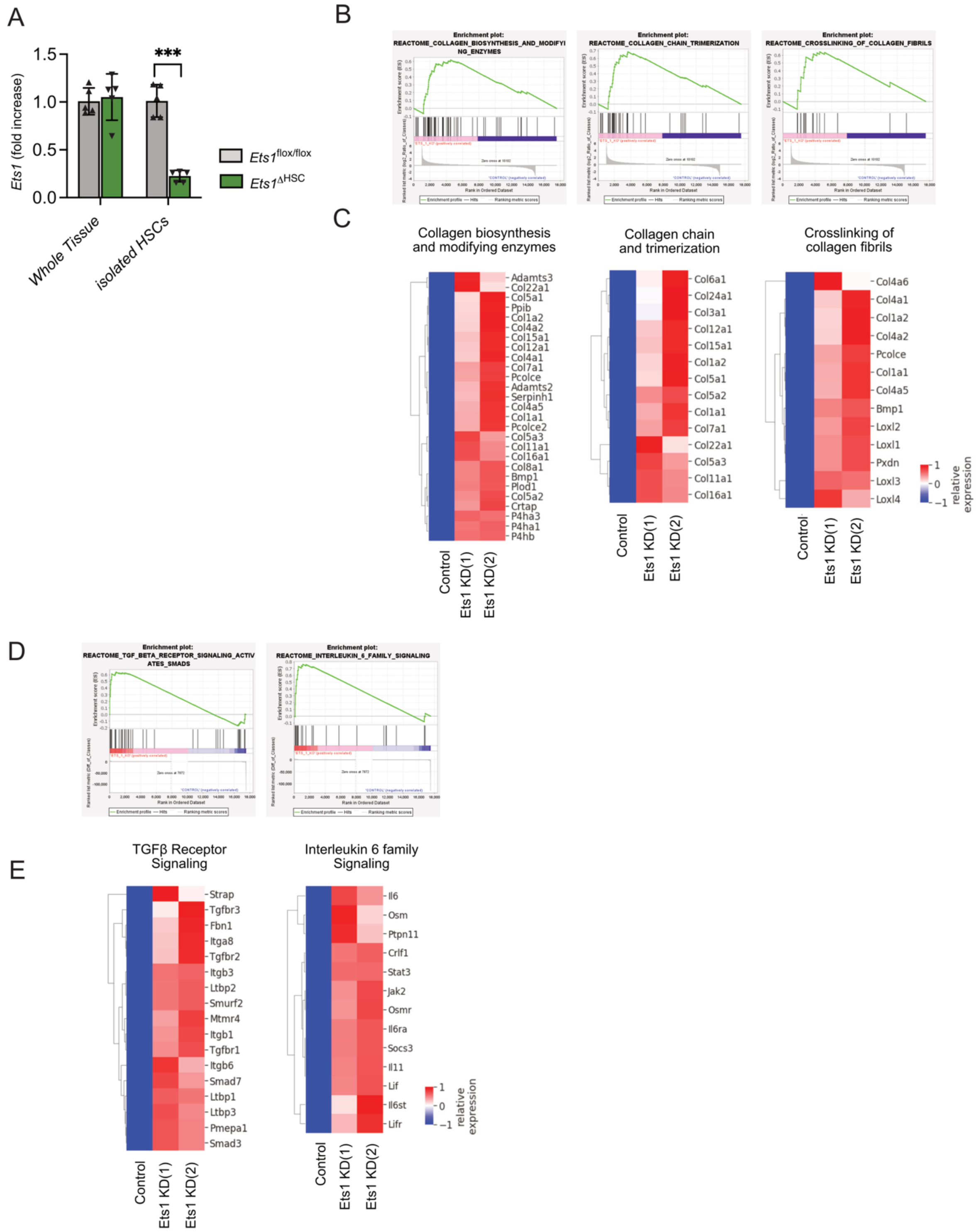

Supplemental Figure 1. Knockdown of ETS1 by shRNA in HSCs resulted in activation of hepatic stellate cell phenotype.

(**A**) Relative mRNA expression of *Ets1* in whole liver tissue and isolated HSCs from *Ets1*$^{flox/flox}$ mice and *Ets1*$^{\Delta HSC}$ mice. (**B-E**) Primary mouse HSCs were infected with control small hairpin RNA (shRNA) (Control) or Ets1-targeting shRNA lentivirus (Ets1 KD), followed by puromycin (5 μg/mL) selection. **(B)** Gene Set Enrichment Analysis (GSEA) plots highlighting the enrichment of specific gene sets related to extracellular matrix (ECM) organization and collagen biosynthesis. **(C)** Heatmaps illustrating the expression levels of genes involved in collagen biosynthesis and modifying enzymes, collagen chain and trimerization, and crosslinking of collagen fibrils. **(D)** GSEA plots highlighting the enrichment of signaling pathways associated with the inflammatory response. **(E)** Heatmaps illustrating the expression levels of genes involved in TGFβ receptor signaling and interleukin-6 family signaling pathways. The color scale represents relative expression levels, with red indicating upregulation and blue indicating downregulation.

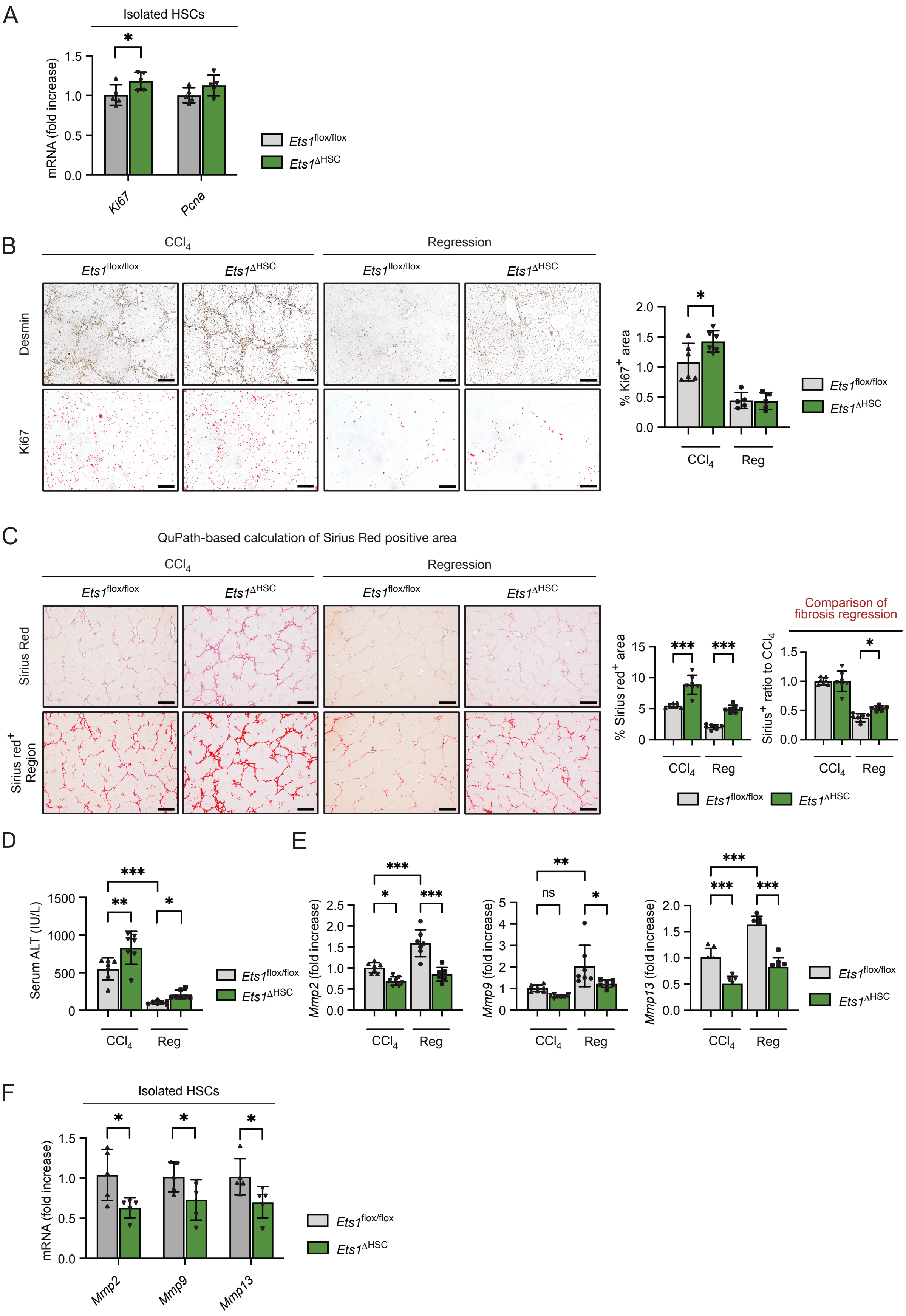

A
Isolated HSCs
mRNA (fold increase)
Ki67
Pcna
Ets1flox/flox
Ets1ΔHSC
B
CCl4
Regression
Desmin
Ki67
% Ki67+ area
CCl4
Reg
C
QuPath-based calculation of Sirius Red positive area
Sirius Red
Sirius red+ Region
% Sirius red+ area
Comparison of fibrosis regression
Sirius+ ratio to CCl4
D
Serum ALT (IU/L)
E
Mmp2 (fold increase)
Mmp9 (fold increase)
Mmp13 (fold increase)
ns
F
Isolated HSCs
Mmp2
Mmp9
Mmp13

Supplemental Figure 2. Deletion of ETS1 in HSCs promotes HSC proliferation and impairs matrix remodeling during liver fibrosis.

(**A**) Expression of proliferation markers (Ki67, Pcna) in isolated HSCs from *Ets1*$^{flox/flox}$ mice and *Ets1*$^{\Delta HSC}$ mice were analyzed by using qRT-PCR **(B-E)** *Ets1*$^{flox/flox}$ mice and *Ets1*$^{\Delta HSC}$ mice (n=7 per group) were administered with either vehicle (corn oil) or carbon tetrachloride (CCl_4) for 6 weeks, followed by cessation for 2 weeks after the last CCl_4 administration. **(B)** Livers from *Ets1*$^{flox/flox}$ mice and *Ets1*$^{\Delta HSC}$ mice were stained for Desmin and Ki67 (scale bar = 200 μm), and Ki67 staining-positive area was calculated as a percentage. **(C)** Livers from *Ets1*$^{flox/flox}$ mice and *Ets1*$^{\Delta HSC}$ mice were stained for Sirius Red (scale bar = 200 μm) to assess collagen deposition. Collagen-positive regions were automatically detected and color-coded (red overlay) using QuPath software, and the percentage of Sirius Red–positive area was calculated by quantitative digital image analysis across multiple random fields (n = 3–5 per sample). **(D)** Serum level of alanine aminotransferase (ALT) was measured. (**E**) Expression of matrix metalloproteinase genes (*Mmp2*, *Mmp9*, and *Mmp13*) in the liver tissues was analyzed by qRT-PCR. (**F**) Expression of matrix metalloproteinase genes (*Mmp2*, *Mmp9*, and *Mmp13*) in isolated HSCs from *Ets1*$^{flox/flox}$ mice and *Ets1*$^{\Delta HSC}$ mice was measured by qRT-PCR. Data are expressed as the mean ± SD; *p < 0.05, **p <0.01, and ***p < 0.001, (A, E) unpaired student's *t*-test; (B–D) one-way ANOVA followed by Tukey's test.

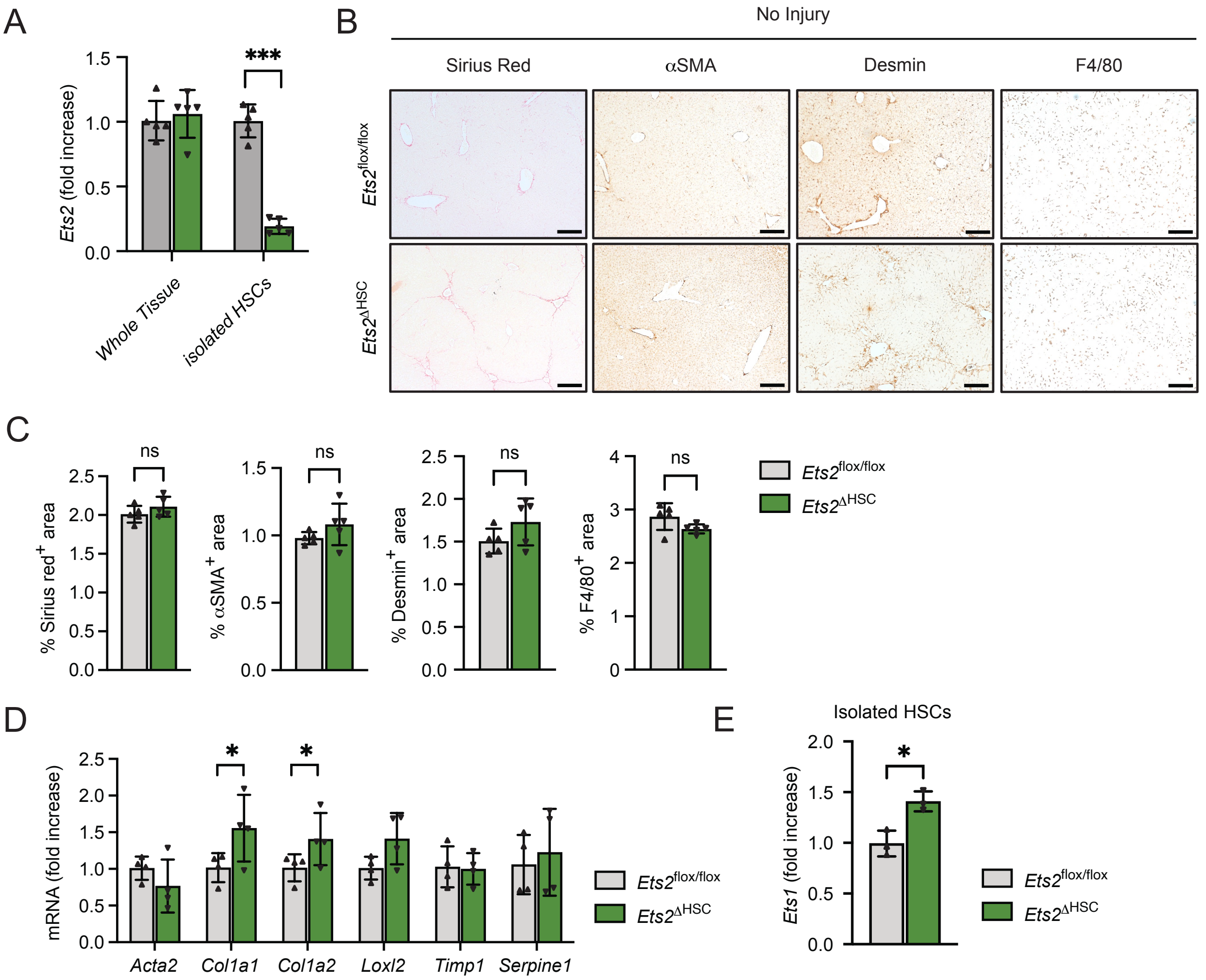

Supplemental Figure 3. Genetic deletion of *Ets2* in HSCs did not cause liver injury in naïve *Ets2*$^{\Delta HSC}$ mice.

(**A**) Relative mRNA expression of *Ets2* in whole liver tissue and isolated HSCs from *Ets2*$^{flox/flox}$ mice and *Ets2*$^{\Delta HSC}$ mice. (**B**) Livers from *Ets2*$^{flox/flox}$ mice and *Ets2*$^{\Delta HSC}$ mice (n=3-5 per group) were stained for Sirius Red, αSMA, Desmin and F4/80. **(C)** Positive area was calculated as a percentage. **(D)** Expression of fibrogenic genes in the liver tissues were analyzed by using qRT-PCR. **(E)** Expression of *Ets1* in activated HSCs isolated from *Ets2*$^{flox/flox}$ mice and *Ets2*$^{\Delta HSC}$ mice were measured by qRT-PCR. Data are expressed as the mean ± SD; *$p < 0.05$ and ***$p < 0.001$, unpaired student's *t*-test.

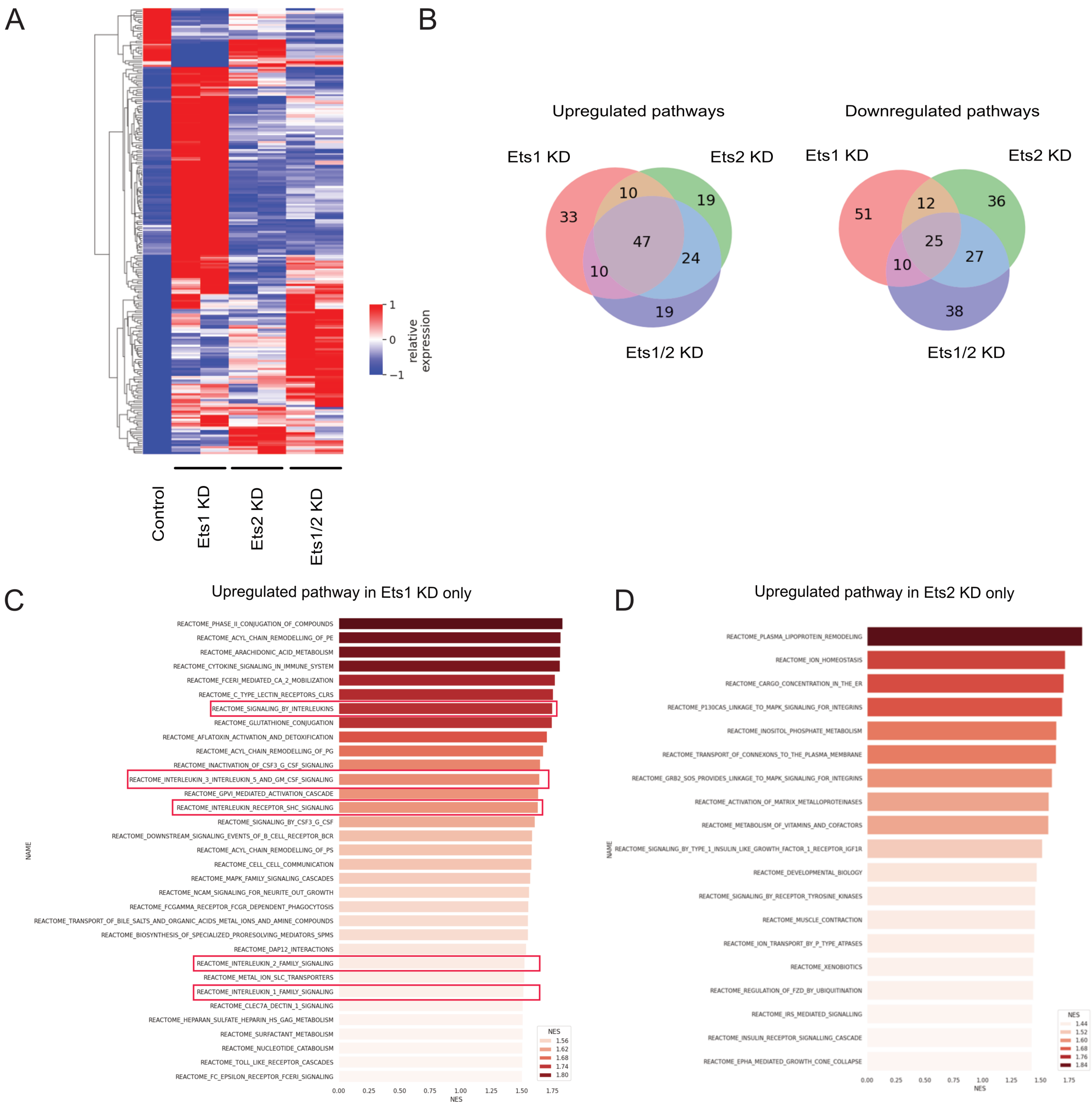

Supplemental Figure 4. Knockdown of Ets1 yielded the most striking phenotype in mouse HSCs.

Primary mouse HSCs were infected with control small hairpin RNA (shRNA) (Control), Ets1-targeting shRNA (Ets1 KD), Ets2-targeting shRNA (Ets2 KD), or Ets1,2-targeting shRNA (Ets1/2 KD) lentivirus, followed by puromycin (5 µg/mL) selection. **(A)** Heatmap analysis of expression of all genes based on RNA sequencing data obtained from control, Ets1 KD, Ets2 KD, and Ets1/2 KD HSCs. Each row represents gene, with the color indicating the level of relative gene expression (blue: low, red: high). **(B)** Venn diagram showing the overlap of upregulated or downregulated pathways in Ets1 KD, Ets2 KD, and Ets1/2 KD cells. Each number represents the genes regulated under specific conditions. **(C-D)** Reactome pathway enrichment analysis of genes uniquely upregulated in **(C)** Ets1 KD, and **(D)** Ets2 KD HSCs. Inflammatory response-related pathways are highlighted with red boxes.

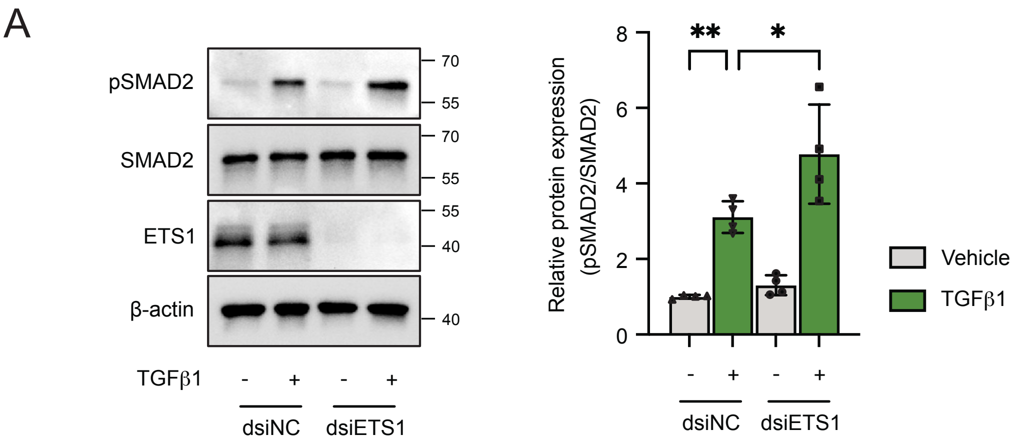

Supplemental Figure 5. ETS1 knockdown enhances TGFβ1-induced SMAD2 phosphorylation in human HSCs.

(A) Western blot analysis of phosphorylated SMAD2 (pSMAD2), total SMAD2, and ETS1 in human HSCs transfected with targeting dsiRNA or dsiNC for 48 h and treated with recombinant human TGFβ1 protein (5 ng/ml) for 24 h. Quantification of relative pSMAD2/SMAD2 protein levels was shown. Data are expressed as the mean ± SD; $^*p < 0.05$, $^{**}p < 0.01$, one-way ANOVA followed by Tukey's test.

A

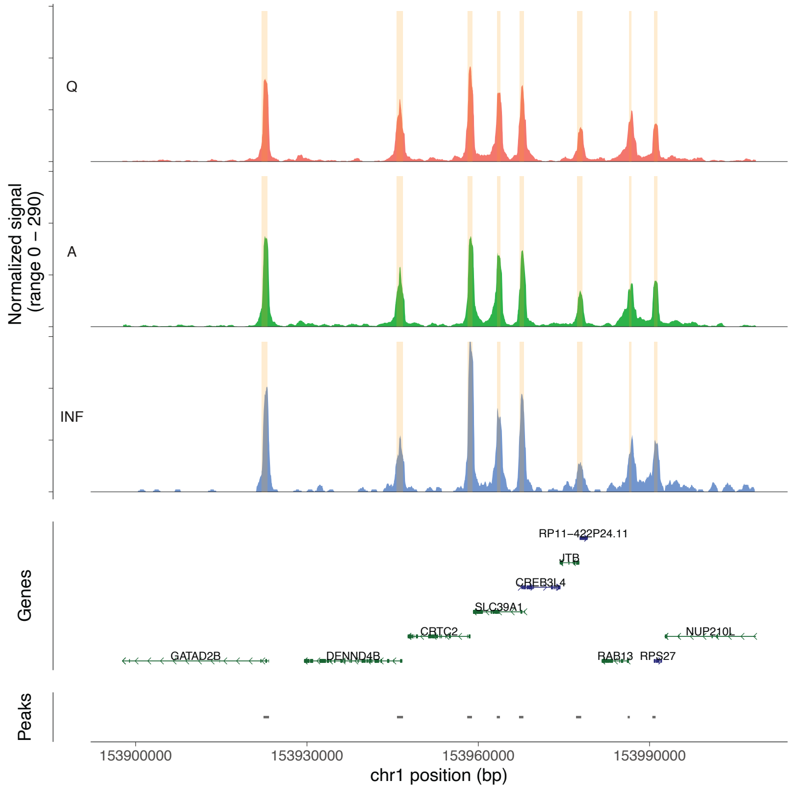

Q
A
INF
Normalized signal
(range 0 – 290)
RP11−422P24.11
LTB
CREB3L4
SLC39A1
CRTC2
NUP210L
GATAD2B
DENND4B
RAB13
RPS27
Genes
Peaks
153900000
153930000
153960000
153990000
chr1 position (bp)

B

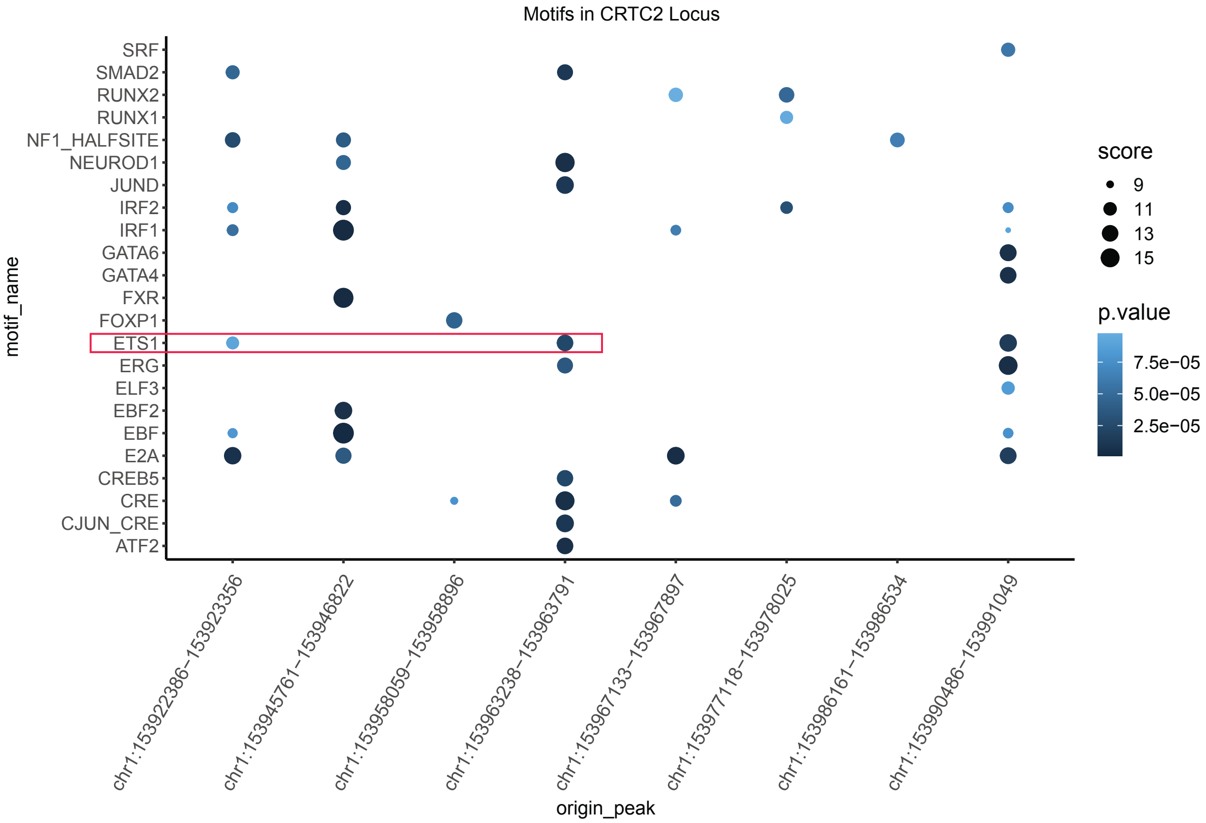

Motifs in CRTC2 Locus
SRF
SMAD2
RUNX2
RUNX1
NF1_HALFSITE
NEUROD1
JUND
IRF2
IRF1
GATA6
GATA4
FXR
FOXP1
ETS1
ERG
ELF3
EBF2
EBF
E2A
CREB5
CRE
CJUN_CRE
ATF2
motif_name
chr1:153922386−153923356
chr1:153945761−153946822
chr1:153958059−153958896
chr1:153963238−153963791
chr1:153967133−153967897
chr1:153977118−153978025
chr1:153986161−153986534
chr1:153990486−153991049
origin_peak
score
9
11
13
15
p.value
7.5e−05
5.0e−05
2.5e−05

Supplemental Figure 6. ETS1 binds to the regulatory element of CRTC2 gene locus

(A) Normalized accessibility plot for the regions surrounding the CRTC2 gene locus on chromosome 1 in human HSCs. The gene annotations and peak locations are shown. **(B)** Dotplots illustrating transcription factors significantly enriched in CRTC2 promoter-linked peaks, as identified by CICERO analysis. Peaks are those with possible regulatory interactions with the core gene promoter based on co-accessibility of peaks. Dot size is scaled to FIMO score, and dot color is scaled to FIMO *p*-value.

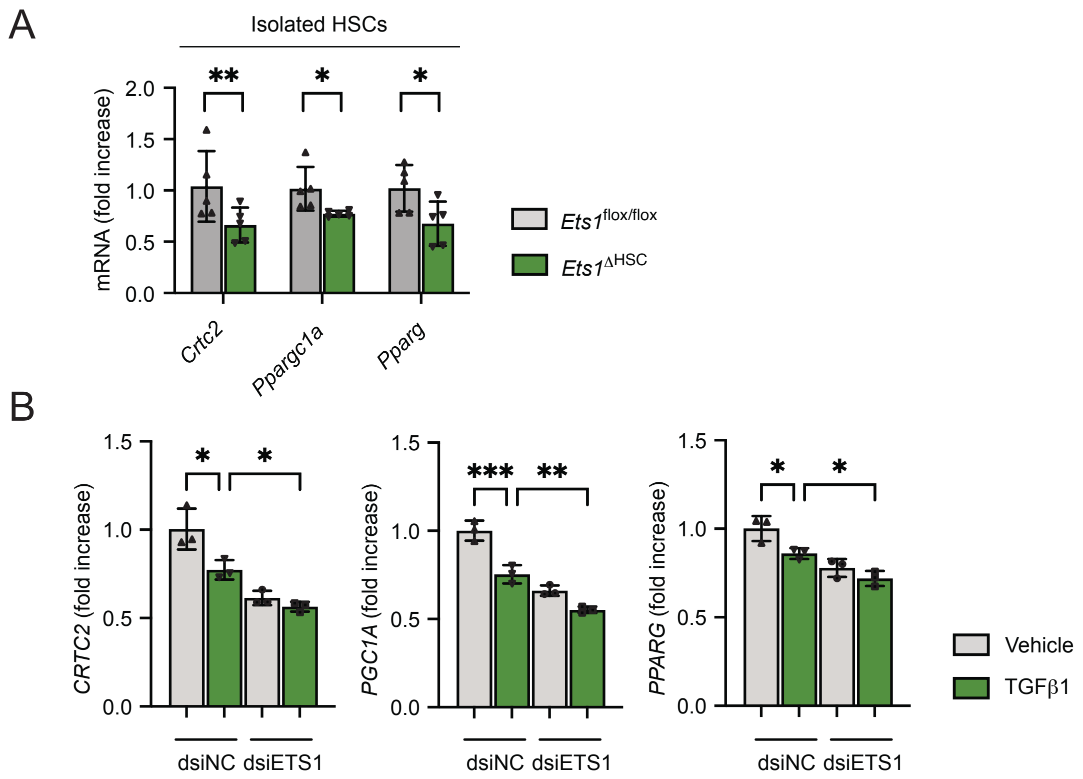

Supplemental Figure 7. ETS1 regulates the CRTC2–PGC1α–PPARγ axis in HSCs.
(A) qRT-PCR analysis of *Crtc2, Ppargc1a,* and *Pparg* expression in isolated HSCs from *Ets1*$^{flox/flox}$ mice and *Ets1*$^{\Delta HSC}$ mice. (**B**) human HSCs were transfected with ETS1 targeting dsiRNA or dsiNC for 48 h and treated with recombinant human TGFβ1 protein (5 ng/ml) for 24 h. mRNA expression of CRTC2, PGC1A, PPARG was measured. Data are expressed as the mean ± SD; *p < 0.05, **p <0.01, and ***p < 0.001, (A) unpaired student's *t*-test; (B) one-way ANOVA followed by Tukey's test.

Supplemental tables and table legends

Table S1. The list of shRNA tested for knockdown of ETS1 and ETS2 in primary mouse HSCs

Symbol	Clone ID	Target Sequence	Vector
ETS1	TRCN0000042640	GCAGACAGACTACTTTCGGAT	pLKO.1
ETS1	TRCN0000042642	GCATCTAGAGATCCTGCAGAA	pLKO.1
ETS2	TRCN0000042649	CCGTCAATGTCAATTACTGTT	pLKO.1
ETS2	TRCN0000218152	CAACACCGTCAATGTCAATTA	pLKO_005
Control	SHC002		pLKO.1

Table S2. The list of dsiRNA tested for knockdown of ETS1 and CRTC2 gene

**Symbol**	**Design ID**
ETS1	hs.Ri.ETS1.13.1
ETS1	hs.Ri.ETS1.13.2
ETS1	hs.Ri.ETS1.13.3
ETS2	hs.Ri.ETS2.13.1
ETS2	hs.Ri.ETS2.13.2
ETS2	hs.Ri.ETS2.13.3
CRTC2	hs.Ri.CRTC2.13.1
CRTC2	hs.Ri.CRTC2.13.2
CRTC2	hs.Ri.CRTC2.13.3
dsiRNA negative control	51-01-14-03

DsiRNA duplexes were purchased from IDT (predesigned dsiRNA). Three different dsiRNA duplexes were tested, and the one with the highest knockdown efficiency (hs.Ri.ETS1.13.2, hs.Ri.ETS2.13.2, hs.Ri.CRTC2.13.2), highlighted in red, was used for further experiments. A dsiRNA negative control was transfected into human HSCs as a control.

Table S3. Primer sequences used for qRT-PCR

Species	Target	Forward Primer (5′ to 3′)	Reverse Primer (5′ to 3′)
Human	*ETS1*	GAGTCAACCCAGCCTATCCAGA	GAGCGTCTGATAGGACTCTGTG
Human	*ETS2*	ACTCCGCCAACTGTGAATTGCC	CCACTGGCATACCTGTTGCTCA
Human	*ACTA2*	CACCATCGGAAATGAACGTTT	GACTCCATCCCGATGAAGGA
Human	*COL1A1*	AAGAGGAAGGCCAAGTCGAG	CACACGTCTCGGTCATGGTA
Human	*COL1A2*	CCTGGTGCTAAAGGAGAAAGAGG	ATCACCACGACTTCCAGCAGGA
Human	*LOXL2*	AGGACATTCGGATTCGAGCC	CTTCCTCCGTGAGGCAAAC
Human	*TGFB1*	TACCTGAACCCGTGTTGCTCTC	GTTGCTGAGGTATCGCCAGGAA
Human	*SERPINE1*	AGTGGACTTTTCAGAGGTGGA	GCCGTTGAAGTAGAGGGCATT
Human	*TIMP1*	AGGTGGTCTCGTTGATTTCT	GTAAGGCCTGTAGCTGTGCC
Human	*PPARG*	GGCTTCATGACAAGGGAGTTT	AACTCAAACTTGGGCTCCATAAAG
Human	*BAMBI*	GGTGCAGGAGCTGACTTCTT	AAGCTGTAGTGCAAACGGGA
Human	*GABRA3*	TGGAAGTGGCACAGGATGGTTC	CGCTTGAGATGGAAGTGGGTTG
Human	*CRTC2*	GGCAGTCTCATTATGGGACACC	TGATGGGCTCTCCATGCTGAAC
Human	*PGC1A*	CCAAAGGATGCGCTCTCGTTCA	CGGTGTCTGTAGTGGCTTGACT
Human	*HPRT*	CCTGGCGTCGTGATTAGTGAT	AGACGTTCAGTCCTGTCCATAA
Mouse	*Acta2*	TGCTGACAGAGGCACCACTGAA	CAGTTGTACGTCCAGAGGCATAG
Mouse	*Col1a1*	GGTCTTGGTGGTTTTGTATTCG	AACAGTCGCTTCACCTACAGC
Mouse	*Col1a2*	TTCTGTGGGTCCTGCTGGGAAA	TTGTCACCTCGGATGCCTTGAG
Mouse	*Des*	CTCGGAAGTTGAGAGCAGAGA	GTGAAGATGGCCTTGGATGT
Mouse	*Timp1*	TCTTGGTTCCCTGGCGTACTCT	GTGAGTGTCACTCTCCAGTTTGC
Mouse	*Loxl2*	TTCTGCCTGGAGGACACTGAGT	TCGGTGATGTCTATCCACTGGC
Mouse	*Tgf b1*	TGATACGCCTGAGTGGCTGTCT	CACAAGAGCAGTGAGCGCTGAA
Mouse	*Serpine1*	CCTCTTCCACAAGTCTGATGGC	GCAGTTCCACAACGTCATACTCG
Mouse	*Il1b*	GGTCAAAGGTTTGGAAGCAG	TGTGAAATGCCACCTTTTGA
Mouse	*Il6*	ACCAGAGGAAATTTTCAATAGGC	TGATGCACTTGCAGAAAACA
Mouse	*Cxcl5*	CCGCTGGCATTTCTGTTGCTGT	CAGGGATCACCTCCAAATTAGCG
Mouse	*Cxcl1*	TCCAGAGCTTGAAGGTGTTGCC	AACCAAGGGAGCTTCAGGGTCA
Mouse	*Bambi*	GCAATTATCGAGGACTGCATGAC	GCGGAACCACAGTTCTTTGGAG
Mouse	*Pparg*	GTACTGTCGGTTTCAGAAGTGCC	ATCTCCGCCAACAGCTTCTCCT
Mouse	*Mmp2*	CAAGGATGGACTCCTGGCACAT	TACTCGCCATCAGCGTTCCCAT
Mouse	*Mmp3*	CTCTGGAACCTGAGACATCACC	AGGAGTCCTGAGAGATTTGCGC
Mouse	*Mmp9*	GCTGACTACGATAAGGACGGCA	TAGTGGTGCAGGCAGAGTAGGA
Mouse	*HPRT*	CTGGTGAAAAGGACCTCTCGAAG	CCAGTTTCACTAATGACACAAACG

Table S4. Antibody list

1st antibody	Supplier	Cat.no	Usage	Concentration
αSMA	Abcam	ab5694	WB, IF, IHC	1:2000, 1:100, 1:200
F4/80	eBioscience	14-4801-82	IHC	1:200
Desmin	Proteintech	60226-1-Ig	IF, IHC	1:500
β-actin	Sigma	A5441	WB	1:4000
PAI-1	Proteintech	13801-1-AP	WB, IF	1:1000, 1:100
Collagen Type 1	Rockland	600-401-103	IF	1:100
Collagen Type 1	Cell Signaling	72026S	WB	1:1000
ETS1	Abcam	Ab124282	WB	1:1000
HNF4α	Invitrogen	MA1-199	IF	1:200
Vimentin	Proteintech	10366-1-AP	IF	1:200
Phospho-SMAD2	Cell Signaling	3108S	WB	1:1000
SMAD2	Cell Signaling	5339S	WB	1:2000
**2nd antibody**	**Supplier**	**Cat.no**	**Usage**	**Concentration**
Goat anti-Rabbit IgG, HRP	Invitrogen	31460	WB	1:2000
Goat anti-Mouse IgG HRP	Invitrogen	31430	WB	1:2000 or 1:5000
Alexa Fluor™ 488 donkey anti-rabbit IgG (H+L)	Invitrogen	A21206	IF	1:200
Alexa Fluor™ 488 goat anti-mouse IgG (H+L)	Invitrogen	A11001	IF	1:200
Alexa Fluor™ 568 donkey anti-rabbit IgG (H+L)	Invitrogen	A10042	IF	1:200
Alexa Fluor™ 594 goat anti-mouse IgG (H+L)	Invitrogen	A11005	IF	1:200
