# Supplementary material for: ETS1 suppresses hepatic stellate cell activation and liver fibrosis
Source: JCI Insight. 2025 Nov 4;10(24):e195242. doi: 10.1172/jci.insight.195242 (PMC12890492; doi:10.1172/jci.insight.195242)

Blots from Figure 4F

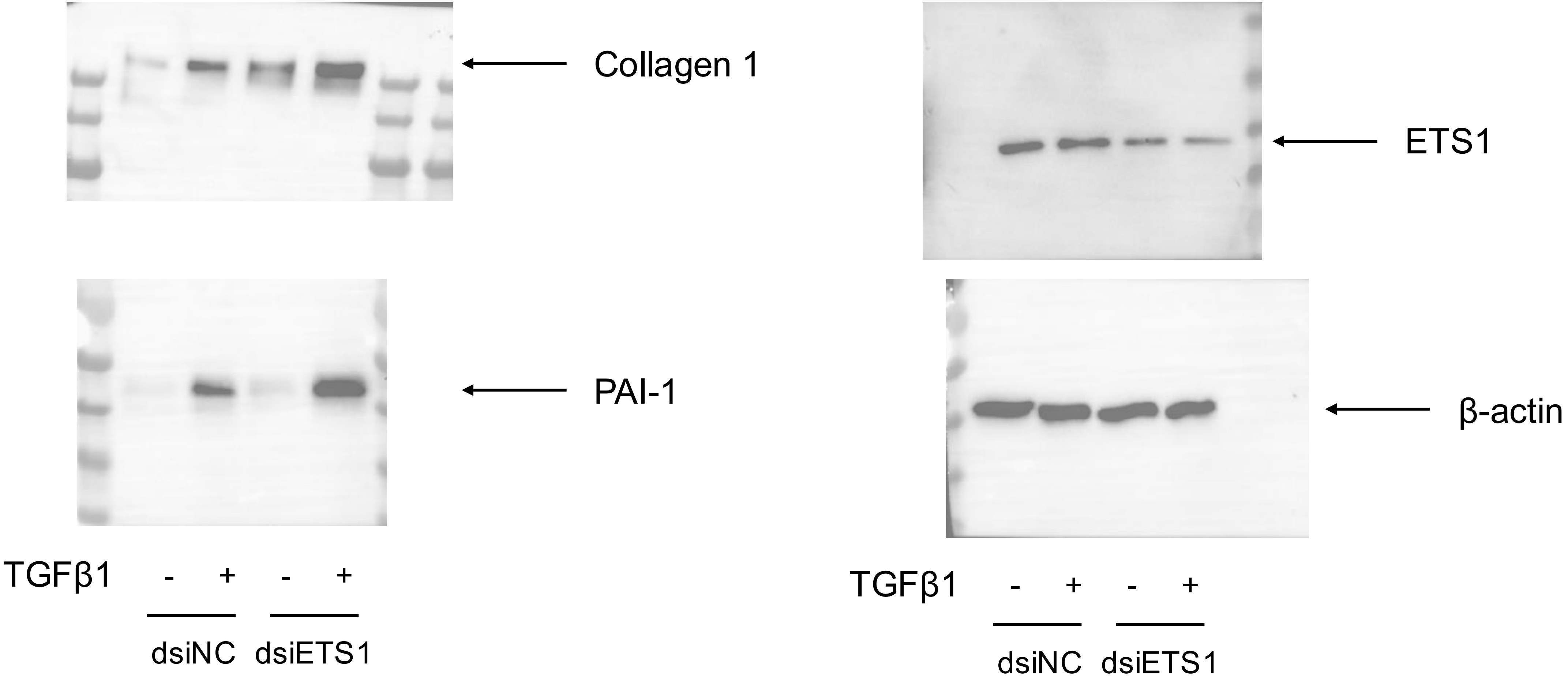

Blots from Figure 5D

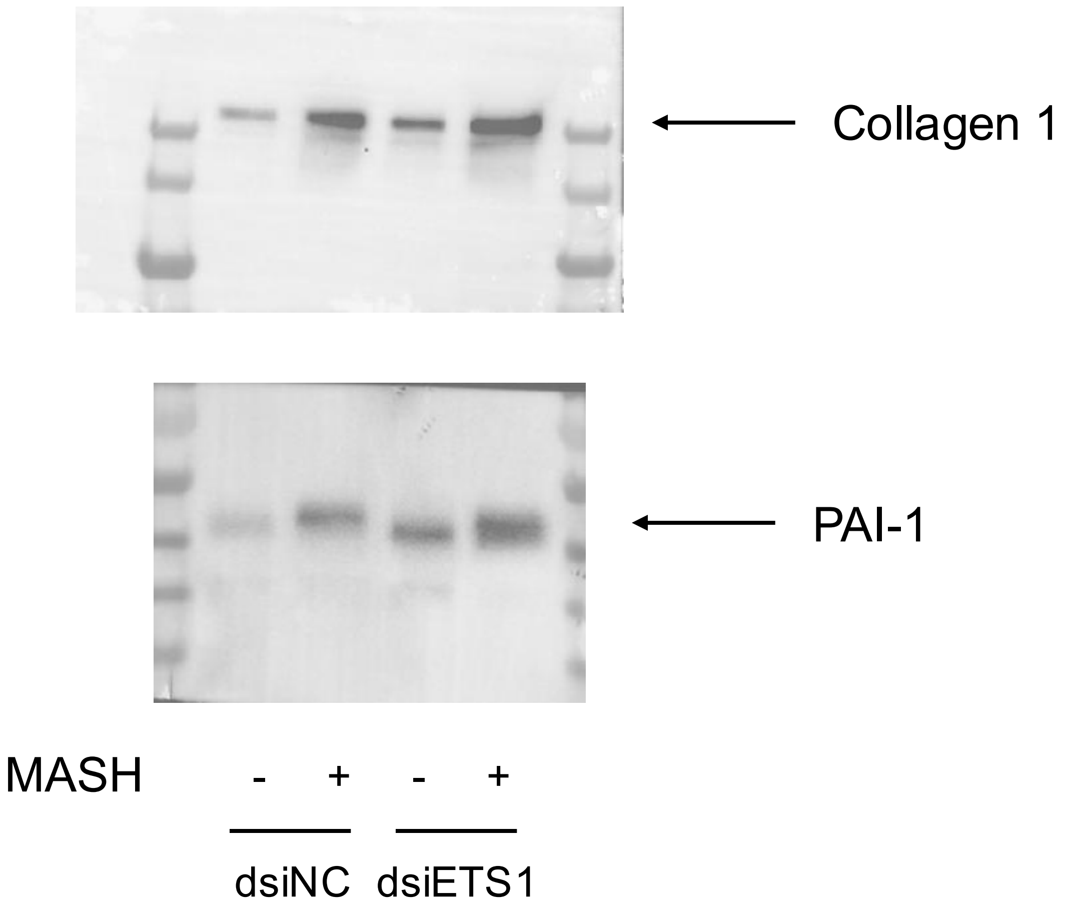


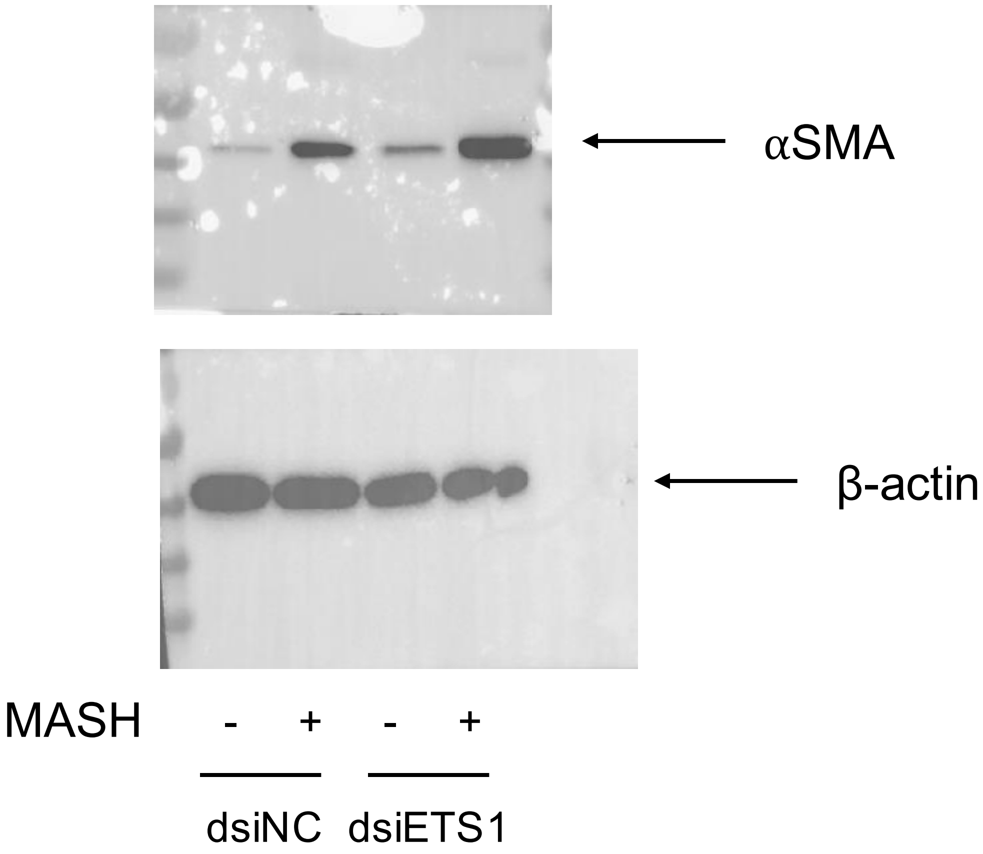

Blots from Figure 6D

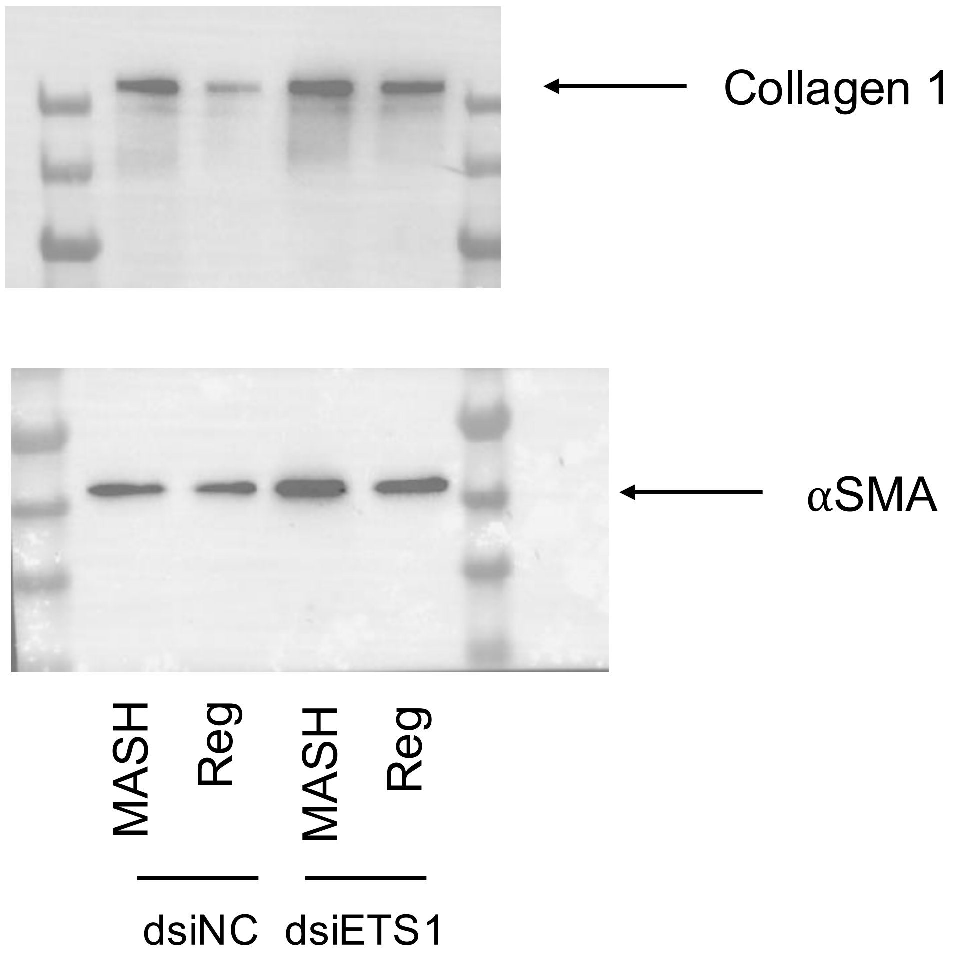


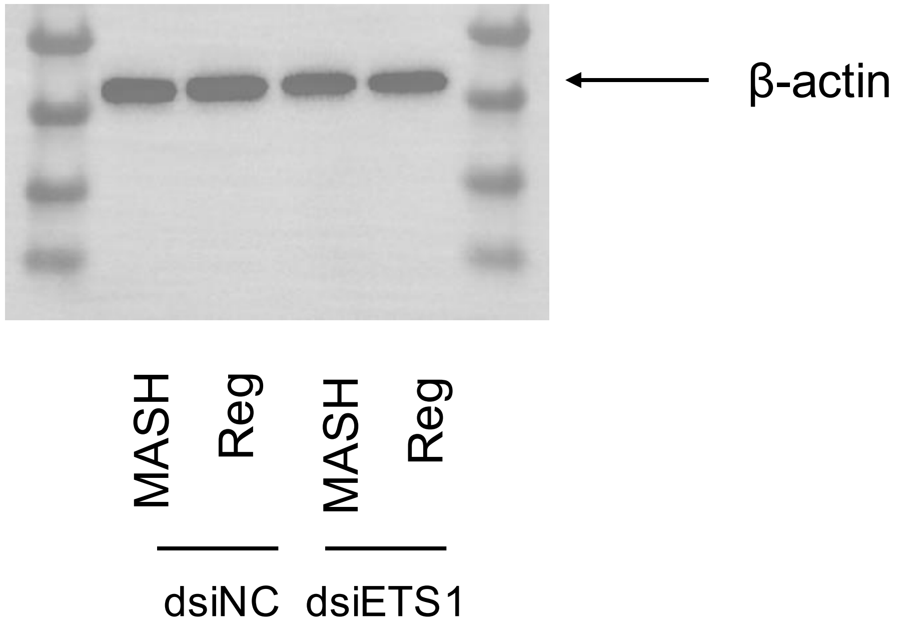

Blots from Figure 7E

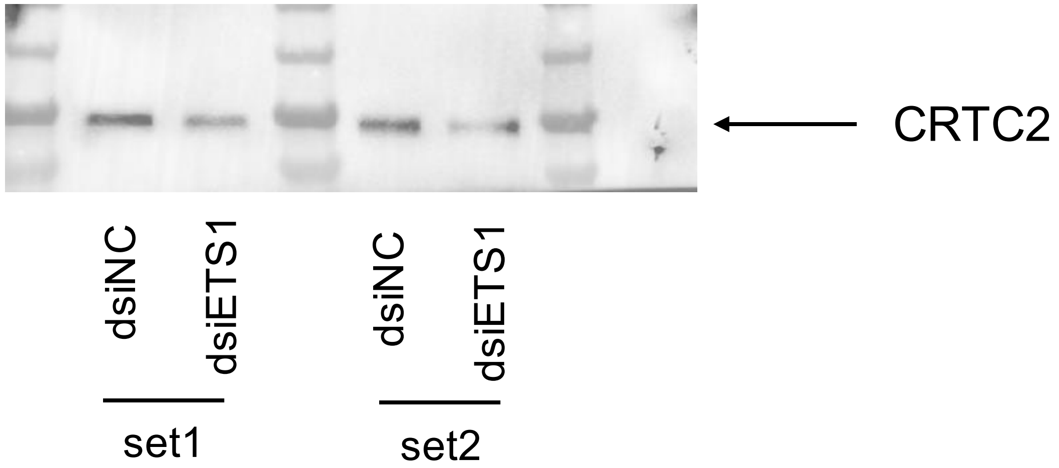


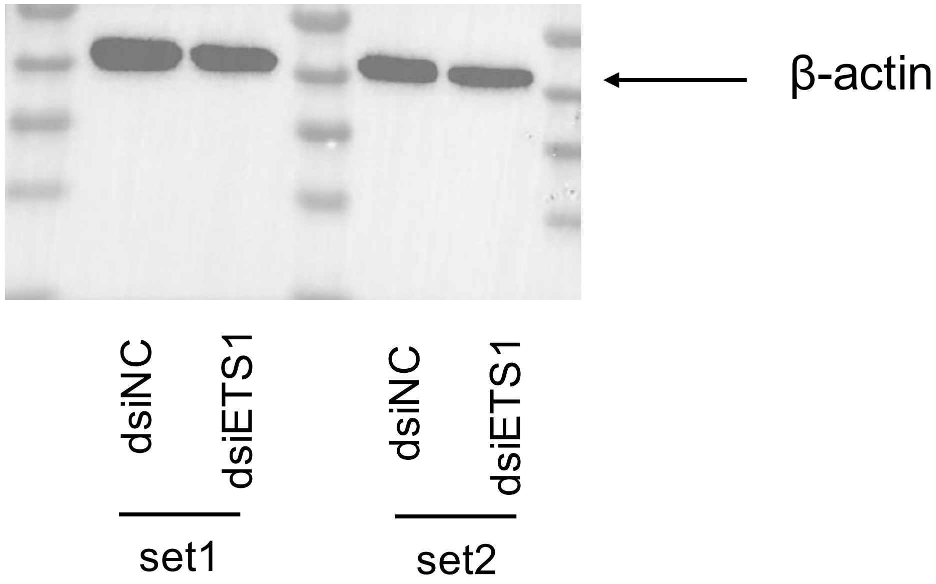

Blots from Figure S5A

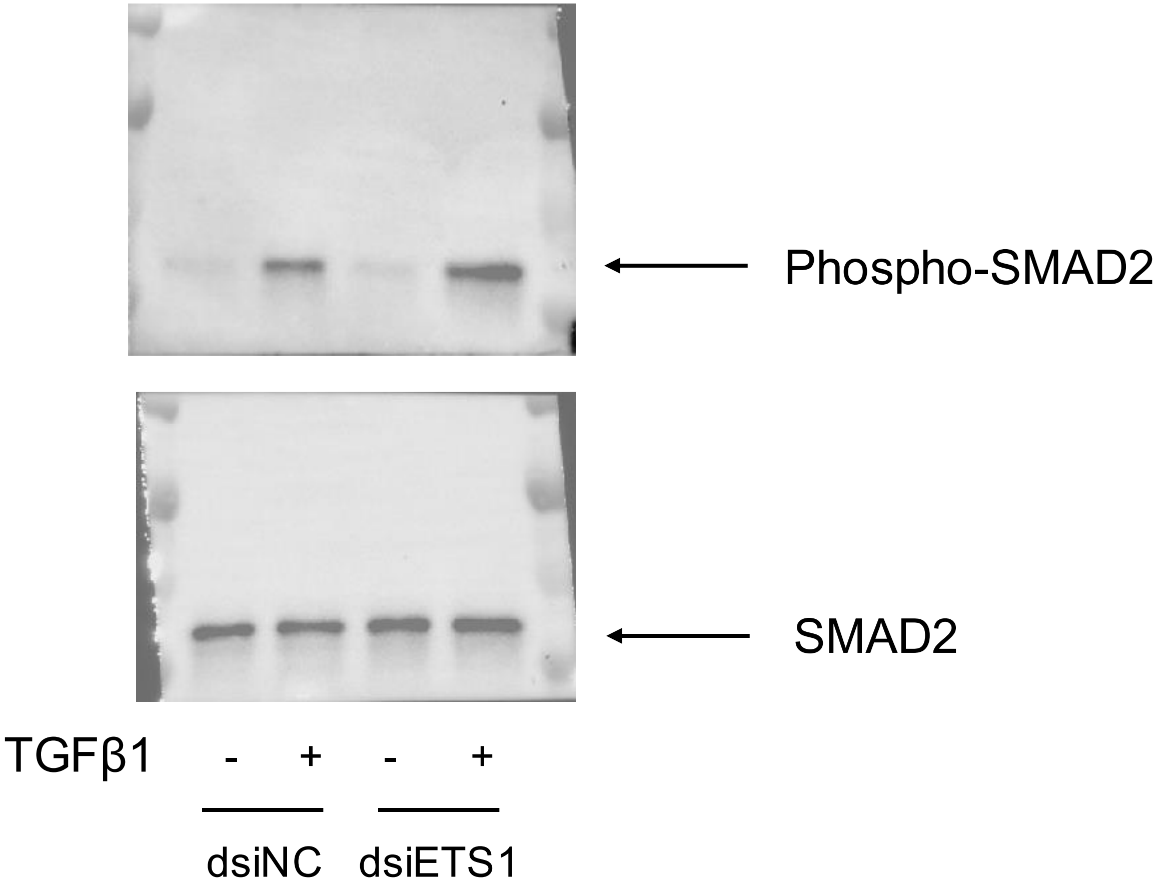


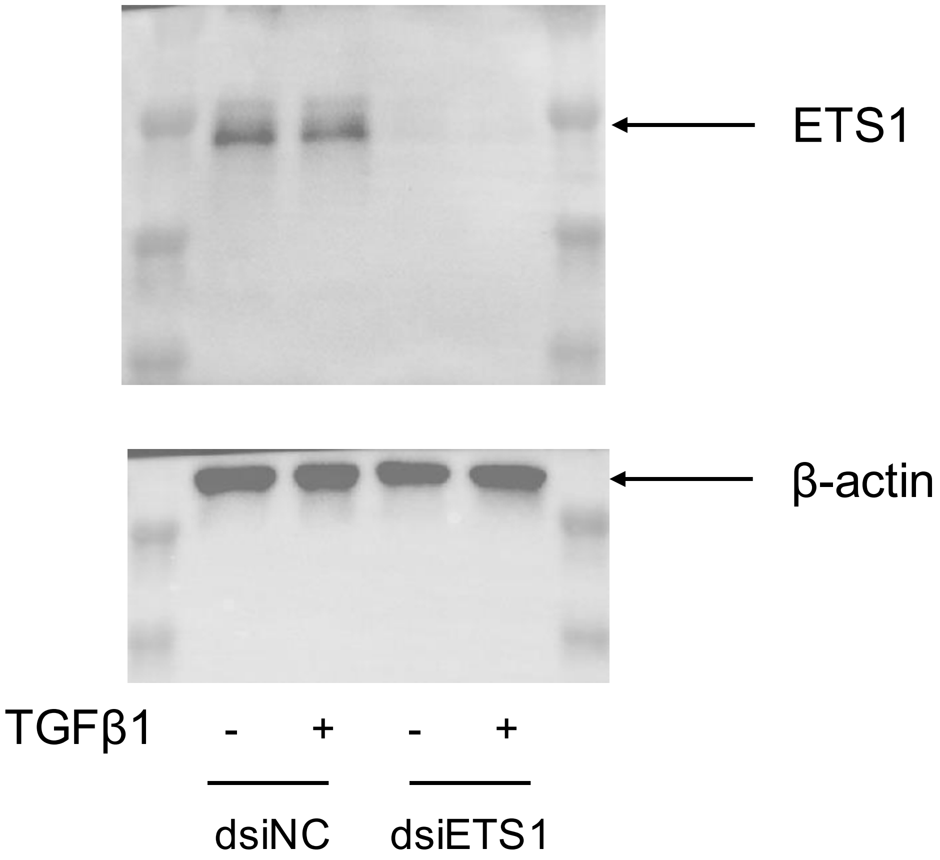

Supplement: Unedited blot and gel images [file jciinsight-10-195242-s085.pdf]
